# Soft Fibrous Syringe Architecture for Electricity‐Free and Motorless Control of Flexible Robotic Systems

**DOI:** 10.1002/advs.202405610

**Published:** 2024-08-19

**Authors:** Chi Cong Nguyen, Trung Thien Hoang, James Davies, Phuoc Thien Phan, Mai Thanh Thai, Emanuele Nicotra, Amr Al Abed, Hien A. Tran, Thanh An Truong, Bibhu Sharma, Adrienne Ji, Kefan Zhu, Chun Hui Wang, Hoang‐Phuong Phan, Nigel Hamilton Lovell, Thanh Nho Do

**Affiliations:** ^1^ Graduate School of Biomedical Engineering Faculty of Engineering and Tyree Institute of Health Engineering (IHealthE) UNSW Sydney Kensington Campus Sydney NSW 2052 Australia; ^2^ College of Engineering and Computer Science VinUniversity Hanoi 100000 Vietnam; ^3^ School of Mechanical and Manufacturing Engineering Faculty of Engineering UNSW Sydney Kensington Campus Sydney NSW 2052 Australia

**Keywords:** motorless, soft robotic arm, soft robotics, soft syringe, teleoperated systems

## Abstract

Flexible robotic systems (FRSs) and wearable user interfaces (WUIs) have been widely used in medical fields, offering lower infection risk and shorter recovery, and supporting amiable human–machine interactions (HMIs). Recently, soft electric, thermal, magnetic, and fluidic actuators with enhanced safety and compliance have innovatively boosted the use of FRSs and WUIs across many sectors. Among them, soft hydraulic actuators offer great speed, low noise, and high force density. However, they currently require bulky electric motors/pumps, pistons, valves, rigid accessories, and complex controllers, which inherently result in high cost, low adaptation, and complex setups. This paper introduces a novel soft fibrous syringe architecture (SFSA) consisting of two or more hydraulically connected soft artificial muscles that enable electricity‐free actuation, motorless control, and built‐in sensing ability for use in FRSs and WUIs. Its capabilities are experimentally demonstrated with various robotic applications including teleoperated flexible catheters, cable‐driven continuum robotic arms, and WUIs. In addition, its sensing abilities to detect passive and active touch, surface texture, and object stiffness are also proven. These excellent results demonstrate a high feasibility of using a current‐free and motor‐less control approach for the FRSs and WUIs, enabling new methods of sensing and actuation across the robotic field.

## Graphical abstract

A novel electricity‐free, motorless, master–slave control methodology—soft fibrous syringe architecture—is presented. This mechanism converts the input motion from the master to the slave without the need for complex mechatronic systems, through hydraulic coupling of artificial muscles, which offers built‐in sensing capabilities. It can be applied to wearable devices and surgical systems where simplicity and miniaturization are necessary.

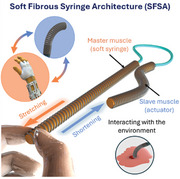

## 1 Introduction

Recently, flexible robotic systems (FRSs) with inherent compliance and adaptability,^[^ ^1^ ^]^ have greatly improved accessibility to the human body, performance of diagnosis, and minimally invasive surgery (MIS).^[^ ^2^ ^]^ Meanwhile, human–machine interactions (e.g., the ability to remotely/virtually manipulate surgical instruments with haptic feedback) have been enhanced by the development of wearable user interfaces (WUIs).^[^ ^1^, ^3^ ^]^ However, achieving high degrees of freedom (DOFs) for these flexible systems often requires multiple actuators, large‐scale, complexity, and costs. Many actuation technologies have been developed for FRSs and WUIs, including motor‐cable actuators,^[^ ^4^ ^]^ shape memory alloys (SMAs), dielectric elastomer actuators (DEAs),^[^ ^5^ ^]^ magnetic actuators,^[^ ^6^ ^]^ and fluidic‐driven actuators,^[^ ^2^, ^7^ ^]^ prizing for their dexterity and safety. While cable‐driven mechanisms, pneumatic soft actuators, magnetic actuators, and others face high friction,^[^ ^8^ ^]^ nonlinear hysteresis,^[^ ^9^ ^]^ high‐cost,^[^ ^10^ ^]^ and complications, hydraulic soft actuators offer low hysteresis, quick responses, miniaturization, high mechanical compliance, high aspect ratios, and precise control.^[^ ^11^ ^]^

Controlling FRSs with implemented motion scaling factors (MSFs) (i.e., output/input motion ratios), especially in critical tasks such as surgery, presents a significant challenge in accurately and effectively translating operators’ inputs to the surgical instruments.^[^ ^11^, ^12^ ^]^ So far, these inputs require a series of mechatronic components (e.g., motors, valves, amplifiers, controllers, encoders) to transmit and perform the tasks.^[^ ^13^ ^]^ This leads to delays and signal degradation, increased complexity and costs, and potential failure. Regarding the MSF, while a small MSF is needed for fine control (e.g., micro‐surgical tasks),^[^ ^14^ ^]^ a larger MSF is necessary for gross positioning and remote target reaching.^[^ ^15^ ^]^ However, implementing them requires additional hardware components (e.g., gearboxes or electronic motion scaling controllers), making the control system even more bulky and complicated.^[^ ^16^ ^]^ In addition, hydraulic‐driven FRSs mainly rely on rigid and bulky driving pressure sources including electrical motors, pumps, and syringes. Recent approaches such as Kirigami‐based^[^ ^14^, ^17^ ^]^ and hydrostatic transmission^[^ ^18^ ^]^ methods have attempted to remove electronic components; however, they still require rigid plungers and seals, involving sliding fictions, limited DOFs, and bulkiness. Other scholars have attempted to remove the rigidity from existing pumps, compressors,^[^ ^19^ ^]^ valves,^[^ ^5^, ^20^ ^]^ and high‐voltage electrical driving sources to achieve flexible and lightweight solutions. Despite advances, these methods still require additional hardware and limited pressure generation—which may be insufficient to activate distal manipulators. In another attempt, Hines et al.^[^ ^21^ ^]^ introduced a dual‐balloon method based on inflated hyperelastic membranes and DEAs. Despite omitting rigid pumps and valves, this structure is difficult to miniaturize given ballooning and requires high‐voltage power sources.

Equipping FRSs with sensors (e.g., position, shape, pressure, and external force^[^ ^1^, ^22^ ^]^) is also crucial to overcoming difficulties in controlling and enhancing the awareness of working environments.^[^ ^23^ ^]^ However, integrating additional sensors with multiple modes into compact and miniaturized flexible robots can be challenging.^[^ ^24^ ^]^ Meanwhile, existing soft sensors exhibit nonlinearities, singular configurations, and nonunique mappings, requiring sophisticated modeling and in‐depth analysis of sensor data.^[^ ^23^, ^25^ ^]^ Methods of adding materials or fluid channels and measuring them by external sensors have been proposed to estimate external strain or force.^[^ ^26^ ^]^ However, additional fluid channels and materials often impact the robot's overall size and mechanical properties. To address this, Shibo et al. proposed a new approach to retrofitting soft fluidic devices using the same channel for actuation that offers self‐sensing ability.^[^ ^27^ ^]^ Nonetheless, this method is restricted to compressible fluids and highly relies on a rigid air tank that requires size adjustments to optimize the system's sensing resolution.

**Figure 1:**
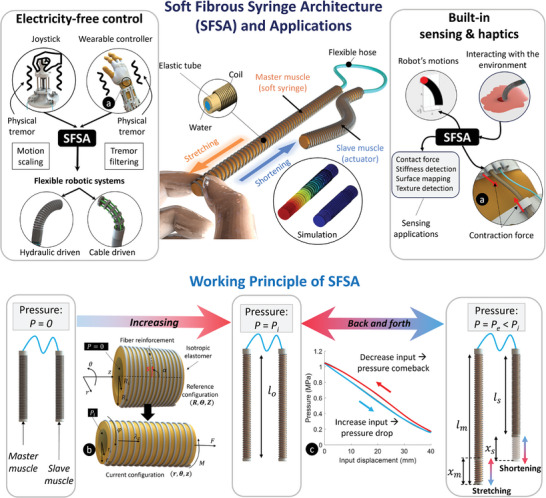
Overview, applications, and simplified working principle of the soft fibrous syringe architecture (SFSA). SFSA is made of two or more connected soft fiber‐reinforced muscles (SFRMs) functioning together. SFSA can be used to control flexible robotic systems without the need to use electric devices such as bulky motors (upper left panel). Based on the change of internal hydraulic pressure of the SFSA, the robot's state and interaction with the environment can be measured (upper right panel). a) Additionally, SFSA can potentially render haptic feedback as a contraction force. b) When hydraulic pressure *P_i_* is introduced, both will elongate to the length *l*_0_. As the continuum mechanical framework is applied, any points in the wall change their coordinates from (*R*,  Θ,  *Z*) to (*r*,  θ,  *z*).^[^ ^31^ ^]^ c) At the pressure *P_i_*, if the master muscle is stretched and then released by a displacement of *x_m_*, the inner pressure will go back and forth, shortening the slave muscle by a displacement of *x_s_* (Movie S1, Supporting Information).

In this work, we introduce a purely mechanical structure coined soft fibrous syringe architecture (SFSA), which offers an electricity‐free and motorless actuation/control method—removing bulky and rigid electronic hardware required by conventional driving sources (e.g., motors, compressors, syringes, valves)—and built‐in sensing abilities for use in FRSs and WUIs (**Figure** **1**). The SFSA has two main parts—master and slave muscles serving as a pliable pump (eliminating coulomb friction^[^ ^18^, ^28^ ^]^) and an active soft actuator, respectively (Figure 1, upper panel). This mechanism can directly convey the master input motions to the slave with highly adaptable MSFs. In addition, this mechanism offers intrinsic, multi‐modal, and tunable sensing abilities for high‐DOF FRSs removing extra sensing components/channels and bulky compressors/tanks.^[^ ^27^ ^]^ While the SFSA can be applied to many types of artificial muscles/actuators (Figure S1, Supporting Information), the most fundamental SFSA (two soft, fiber‐reinforced muscles—SFRMs) is focused in this report as it aligns closely with the stringent requirements for medical devices with remarkably high aspect ratios, high uniformity, simplicity of fabrication methods, and miniaturization,^[^ ^29^ ^]^ compared to other existing fiber‐reinforced soft actuators.^[^ ^30^ ^]^

## 2 Results

### 2.1 Conceptual Design and Operating Principle of SFSA

#### 2.1.1 Introducing the SFSA

The SFSA's SFRM comprises an inner rubber tube radially reinforced by an external helical coil made of inextensible fibers. Details of SFSA fabrication are shown in Note S1 (Supporting Information). The helical coil has a fiber‐direction angle, which is defined by the angle between the fiber and the longitudinal axis (Figure 1b). When a single SFRM is supplied a hydraulic pressure from 0 to *P_i_*, its inner rubber tube is forced to expand in all directions. However, due to the radial constraint caused by the outer helical coil, this rubber tube is unable to expand fully radially from *R* to *r*, yet freely extends along its longitudinal axis from *L* to *l* while untwisting the coil, resulting in an end‐to‐end twisting angle φ (Movie S1, Supporting Information). At this level of initial pressure *P_i_*, the SFRM is holding elastic potential energy and tends to shrink back to its original state. In an SFSA, if one of the SFRMs (the master muscle) is further stretched by a displacement *x_m_* (or a new length *l* + *x_m_*), its fluid chamber volume is increased, and the master elastic tube is destressed in the radial direction. Due to the pressure drop from *P_i_* to *P_e_* (*P_e_* < *P_i_*) in the master muscle (Figure 1c); the other muscle (the slave muscle) will be shortened by a distance *x_s_* (or a new length *l* − *x_s_*). It is noted that the change of the fluid pressure can be measured by an external pressure sensor which will be employed for sensing capability. The ratio *x_s_*/*x_m_* is defined as the MSF or the motion sensitivity of the SFSA, while the force sensitivity is specified by the ratio *F_o_*/*F_i_* between the external input force applied to the master muscle and the output force generated by the slave muscle. In addition, we defined the ratio between the change of inner pressure and input force (*P_i_* − *P_e_*)/*F_i_* as the sensing sensitivity of the SFSA. The three sensitivities—motion, force, and sensing—are all tunable in several ways such as changing the SFSA's internal pressure or physical configuration. This means that the sensitivities can be either tuned before each application or in situ. Notably, the mechanism mentioned above is the most fundamental form of the SFSA, while it could have different types of fiber‐reinforced artificial muscles (e.g., structures, sizes, and materials) (Figure S1, Supporting Information) and a different number of muscles for both sides. Potential applications of the SFSA on other SFRMs will be discussed in Section 3 of this manuscript.

In the fundamental SFSA, the master muscle not only possesses a fibroid structure that proves highly advantageous for the development of FRSs and WUIs—but it also functions as a pliable pump or soft syringe for the slave muscle. The soft syringe, depicted as the master muscle in Figure 1, functions on a contrary principle compared to a conventional rigid syringe, eliminating the necessity for a plunger. Instead of using external pushing force to pump fluid or induce the change of hydraulic pressure, our soft syringe is supplied by a strain to reduce the pressure. Regarding slave muscles, thanks to having a fibroid morphology, they can be formed into complex continuum shapes while changing their constraints and structures. Another significant novelty of the SFSA compared to other available actuation mechanisms is that it can simultaneously provide both actuation and built‐in sensing capacities without requiring any bulky and rigid compressors or tanks.^[^ ^27^ ^]^ This factor can increase the optimization for the design of soft robotic systems (SRSs), where the need to affix additional sensors to the end‐effector of SRSs can be eliminated. In existing teleoperated systems such as robotic catheters, extra strain, and force sensors are required to monitor their instantaneous bending motion and tool‐tissue contact force. In contrast, a soft robotic catheter using SFSAs (see next Section 2.3) can provide self‐sensing ability without the need for additional sensors, which can further minimize the size and bulkiness of the overall device.

#### 2.1.2 Analytical Modeling of the SFSA

We developed a kinematic model for this fundamental SFSA to understand its behaviors and predict the interactions between the motions of the master and slave muscles. In this model, we consider the wall of the single soft actuator of the SFSA as a composite material with an isotropic hyper‐elastic matrix and anisotropic embedded fiber (Figure 1b). The details of key assumptions and derivations are shown in Note S2 (Supporting Information). Briefly, under applied pressure, the possible extension, expansion, and twisting deformations of the SFRM are represented by the deformation gradient ***F***:^[^ ^31^ ^]^

(1) $$F=\left(\begin{array}{ccc} \frac{R}{r\lambda_{z}} & 0 & 0\\ 0 & \frac{r}{R} & \frac{r\varphi }{L}\\ 0 & 0 & \lambda_{z} \end{array}\right)$$

where *R* and *r* (mm) are the radial coordinates in the reference and current configurations, respectively, λ_*z*_ =  *l*/*L* is the axial stretching ratio when the SFRM longitudinal length changes from *L* → *l* (mm), and φ (rad) denotes the rotational angle between one end to the other end. When the SFRM deforms, the fiber change is described as the deformed fiber direction ***s***:

(2) $$s=\mathrm{FS}=\left[\begin{array}{c} 0\\ \frac{r}{R}\sin\left(\alpha \right)+\frac{r\varphi }{L}\cos\left(\alpha \right)\\ \lambda_{z}\cos\left(\alpha \right) \end{array}\right]$$

where ***S*** = [0 sin(α) cos (α)]^*T*^  is defined as a unit vector tangent to the helical coil in the SFRM's initial configuration, with α (rad)—the angle between the fiber and the longitudinal axis. We used a basic incompressible Neo–Hookean model for the elastomeric tube and a linear spring model for the fibered layer to describe the overall strain energy through the superposition of elastomer and fiber energy:

(3) $$\Psi =\Psi_{tube}+\Psi_{fiber}=\frac{\mu_{1}}{2}\left(I_{1}-3\right)+\frac{\mu_{2}}{2}{\left(\sqrt{I_{4}}-1\right)}^{2}$$

where μ_1_ and μ_2_ (MPa) are the material properties of the SFRM; *I*_1_ =  *tr*(***FF** ^T^*) and *I*_4_ =  ***s***. ***s*** represent for the first and fourth tensor invariants, respectively. Based on that, we applied the Cauchy equilibrium equation to identify the current SFRM configuration: ∇. ***σ *** = *** *** 0, where $\sigma =\left[\begin{array}{ccc} \sigma_{rr} & \sigma_{r\theta } & \sigma_{rz}\\ \sigma_{\theta r} & \sigma_{\theta \theta } & \sigma_{\theta z}\\ \sigma_{zr} & \sigma_{z\theta } & \sigma_{zz} \end{array}\right]=\frac{\partial \Psi }{\partial F}\;F^{T}-pI$ is the Cauchy stress with *p* is a hydrostatic pressure^[^ ^32^ ^]^ and ***I*** is the identity matrix. Applying the boundary conditions for the first hydrostatic equilibrium equation and a variable change from *r*‐to‐*R* yields:

(4) $$P=\int_{R_{i}}^{R_{o}}\frac{R}{\left(R^{2}-R_{i}^{2}+\lambda_{z}r_{i}^{2}\right)}\left(\sigma_{\theta \theta }-\sigma_{rr}\right)dR$$

where *P* (Pa) is the internal pressure applied to the SFRM, *R_o_*, *R_i_*, *r_o_*, and *r_i_* are respectively outer and inner radius of the reference and current configurations; and the subtraction σ_θθ_ − σ_*rr*_ can be derived from the Cauchy stress. Since there are no external axial forces or external axial moments applied to the single SFRM, the axial load *N* (N) and the axial moment *M* (N.mm) can be expressed as follows:

(5) $$N=2\pi \int_{r_{i}}^{r_{o}}\sigma_{zz}rdr=P\pi r_{i}^{2}$$

(6) $$M=2\pi \int_{r_{i}}^{r_{o}}\sigma_{\theta z}r^{2}dr=0$$

Equations (4), (5), and (6) are the key equations of a single muscle describing the relationship of three kinematic quantities (end‐to‐end rotation φ, axial stretch *l*, and internal radius *r_i_*) and three kinetic quantities (internal pressure *P*, twisting moment *M*, and axial load *N*), which fully defines its shape and loading. Thereafter, three static boundary conditions were applied for the closed‐loop system of SFSA. First, the pressures inside the master and slave muscles are equal at the equilibrium state. Extending from Equation (4) for two muscles, yields:

(7) $$\begin{array}{ccc} & & \int_{R_{i}}^{R_{o}}\frac{R_{a}}{\left(R_{a}^{2}-R_{i}^{2}+\frac{l_{a}}{L}r_{ia}^{2}\right)}\left(\sigma_{\theta \theta a}-\sigma_{rra}\right)dR_{a}\\ & & \;=\int_{R_{i}}^{R_{o}}\frac{R_{b}}{\left(R_{b}^{2}-R_{i}^{2}+\frac{l_{b}}{L}r_{ib}^{2}\right)}\left(\sigma_{\theta \theta b}-\sigma_{rrb}\right)dR_{b} \end{array}$$

where *r_ia_* and *r_ib_* denote the inner radii, and σ_θθ*a*_, σ_*rra*_, σ_θθ*a*_, and σ_*rrb*_ are the stresses of the two muscles at the equilibrium state; *R_a_* and *R_b_* are radial in the reference configurations of the two muscles. Second, the volume inside the master muscle increases, and the liquid volume technically moves from the slave muscle to the master muscle, hence the volume changes in these two muscles are equal:

(8) $$l_{a}r_{ia}^{2}-l_{P}r_{iP}^{2}=l_{P}r_{iP}^{2}-l_{b}r_{ib}^{2}$$

Since the slave muscle is free, the last two equations are similar to Equations (5) and (6), but apply to the slave muscle:

(9) $$\pi \int_{R_{i}}^{R_{o}}\left(2\sigma_{zzb}-\sigma_{rrb}-\sigma_{\theta \theta b}\right)\frac{LR_{b}}{l_{b}}dR_{b}=0$$

(10) $$2\pi \int_{R_{i}}^{R_{o}}\frac{L\sigma_{\theta zb}r_{b}R_{b}}{l_{b}}dR=0$$

Solving Equations ((7), (8), (9), (10)) yields the values of four unknown parameters *l_b_*, *r_ib_*, *φ_b_*, and *r_ia_*, that define the output behaviors of the slave muscle from the input conditions of the master muscle.

### 2.2 Characterizations of the SFSA

#### 2.2.1 Validation of the Model and System Limited Point

**Figure 2:**
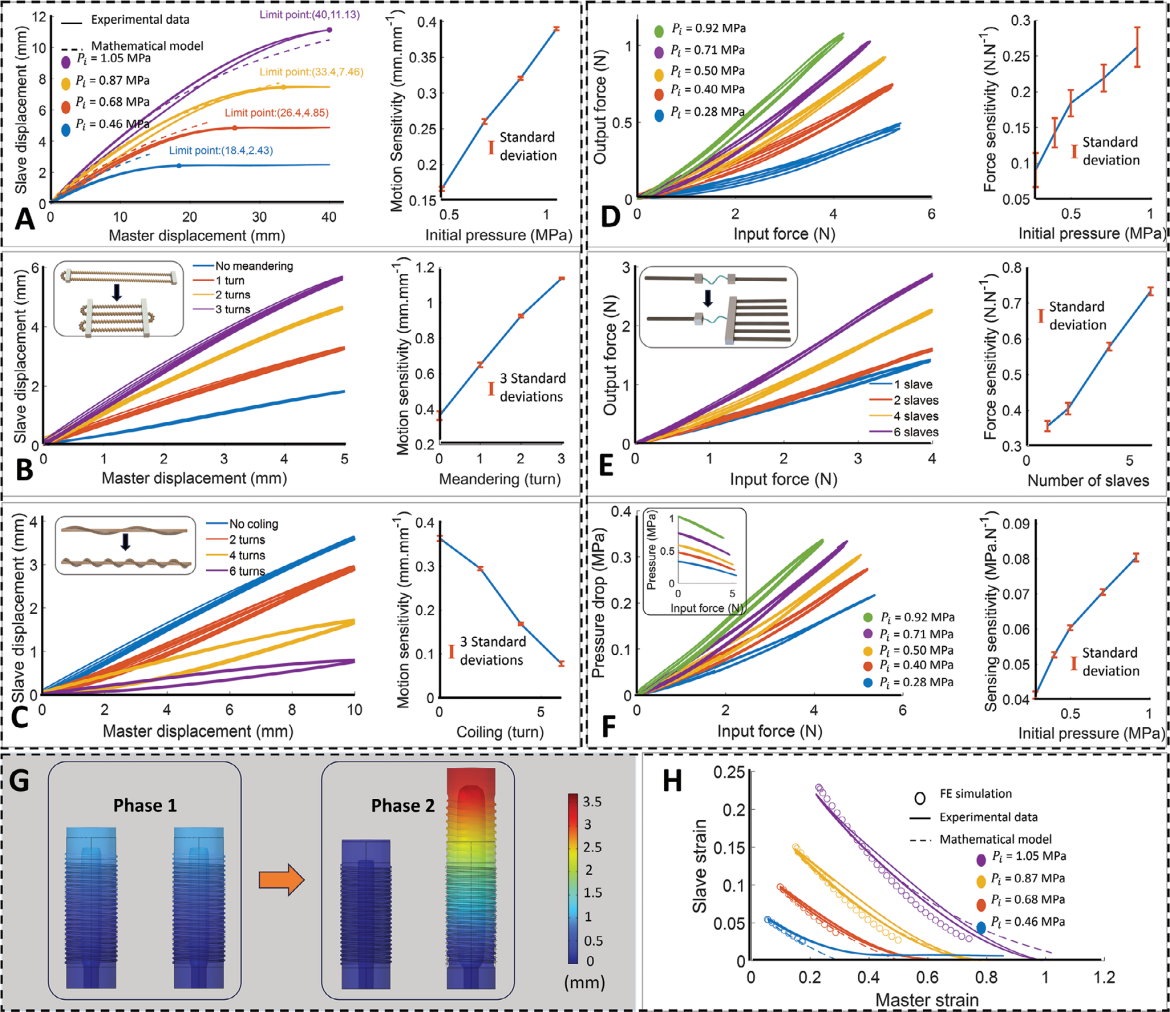
Motion (top left panel) and force (top right panel) characterizations and FE simulation (bottom panel) of the SFSA: A) Mathematical model and experimental data of the master and slave muscle's motion with the system's limit points (left). Computed motion sensitivities against the initial pressure (right). B) Input and output motions of the SFSA when meandering the SFSA's master muscle from zero to three turns (left). Computed motion sensitivities against the initial pressure (right). C) Input and output motions of the SFSA when coiling the SFSA's master muscle around a rubber tube from zero to six turns (left). Computed motion sensitivities against the initial pressure (right). D) Experimental data of the force characterization for the SFSA with five distinct initial pressures (left). Computed force sensitivities against the initial pressure (right). E) Input and output forces when adding slave muscles from one to six muscles (left). Computed force sensitivities against the initial pressure (right). F) Calculated pressure drops from experimental data versus the input force when the slave muscle end is fixed (Figure S8, Supporting Information) (left). Computed force sensitivities against the initial pressure (right). G) Finite element (FE) simulation results of two small segments of the SFSA's muscles in two operating phases. H) Comparisons of FE simulation results versus experimental data and mathematical model in terms of strain.

To characterize the fundamental SFSA and validate the developed mathematical model, we conducted an experiment using two connected SFRMs (Ø2.5 × 50 mm) (Table S1, Supporting Information). Details of experimental configurations are described in Figure S5 (Supporting Information) (Note S3, Supporting Information) and Section 4. We initially validated the model for the single SFRM by recording its strain against the input pressure, yielding a well‐fitted relationship (Figure S3A, Supporting Information). Then, we applied four different initial pressures to observe the behavior of the SFSA. The change in the SFSA's internal pressure during the activation period was measured by external pressure sensors (Figure S1F, Supporting Information). The results shown in **Figure** **2**A (left) reveal good agreements between our mathematical model and the experimental data when the initial pressure is high, specifically in the case of *P_i_* = 1.05 and *P_i_* = 0.87 MPa. By contrast, as the initial pressure decreases, the model's accuracy reduces noticeably, as evidenced by the case of *P_i_* = 0.46 MPa. These discrepancies can be attributed to two factors: the presence of air bubbles during the fabrication process and the interaction effects, such as sliding motions between the coil and elastomeric tube. At higher pressures, the air bubbles have been compressed mitigating their effects on the SFSA performance, while the elastomeric tube is strained more against the coil, resulting in a higher sliding friction that prevents relative movements between them. From the mathematical model, we discerned a critical threshold which, once surpassed, the equations become infeasible to solve. In other terms, the boundary conditions are violated. Therefore, no results are shown beyond this point. We also designated this threshold as the system's limit point and the range from 0 to this point as the operating range of the SFSA. We also identified these limit points in the experimental data by fitting the data with an eighth‐order curve. The clear plateauing of experimental output motion past these thresholds evidences the physical relevance of our proposed model, with boundary conditions rooted in real‐world mechanics. It is worth highlighting that the limit points of the SFSA jump when the initial pressure increases. In other words, with a higher initial pressure, the SFSA has a wider operating range. This trend is reflected in both the modeled response and the experimental data, further demonstrating the physical relevance of our model. Additionally, the predictions of limit points at higher pressure were more accurate than at lower pressure.

Figure 2A (left) confirms that the motion sensitivity of the SFSA is tunable as output motion is increased when the initial pressure increases to input motion. If we consider the short range (<15 mm) of the master muscle's displacement or equivalent to 30% of the muscle's initial length, the experimental data unveils predominantly linear relationships with low hysteresis between input and output displacements (Figure S3B, Supporting Information). Within this range, motion sensitivities were calculated for each initial pressure as the results shown in Figure 2A (right). The motion sensitivity at the highest initial pressure (0.39 mm mm^−1^) is ≈2.3 times higher than that of the lowest initial pressure (0.17 mm mm^−1^). However, as we extended our examination to longer displacement ranges, a noticeable increase in hysteresis became evident (Figure 2A (left)). In effect, the hysteresis of the system primarily stems from the viscoelastic properties of the elastomer—the rubber tube. Specifically, the elastomer combines viscous (fluid‐like) and elastic (solid‐like) properties. When stressed, it experiences both elastic deformation (recoverable) and viscous dissipation (energy loss or non‐recoverable).^[^ ^33^ ^]^ During pressurization, the elastomer undergoes both recoverable expansion and some non‐recoverable deformation within the helical coil—the fiber‐reinforced layer. This internal energy dissipation during pressurization results in a difference in the pressure required for contraction compared to expansion,^[^ ^29^, ^34^ ^]^ leading to the observed hysteresis loops in Figure 2A (left). As can be seen from Figure S3E (Supporting Information), the active pressure range (i.e., the pressure value ranging from the starting point to the point where its value flattens—the system's limit point) is larger for the higher initial pressure compared to the lower one. As a result, the hysteresis loops of higher initial pressure exhibit greater enclosed areas than those observed for lower initial pressure.

#### 2.2.2 Durability and Response Latency Test

To further examine the impacts of hysteresis and material properties on the SFSA performance, durability, and response latency tests of the SFSA were carried out using the same setup as the above motion characterization (Figure S5, Supporting Information). As discussed above, the hysteresis creates latency in the SFSA system, however, the response latency is insignificant within a small range of motions (such as 1 mm of output motion). As evidence, Figure S3C (Supporting Information) shows the remarkably low response latency of the output motion to the input motion (frequency of 0.2 Hz), which is only 0.031 ± 0.017 s. The durability test was conducted for seven consecutive days (in 5400 s for each day with a frequency of 0.2 Hz) with the results and computed shrinkage rates presented in Figure S3D (Supporting Information). It is worth noting that the constant initial pressure of 0.7 MPa was used and maintained and the prototype was left under pressurization over the testing period. The shrink rate exhibited a significant increase, rising from a minimal 1.1% to a pronounced 7.63% over the testing week. The growth of the shrinkage rates can be attributed to the materials of the muscles, where the helical coil and rubber tube are hyperelastic components affected by fatigue after performing multiple periodic cycles of stretching over a long period. While future research on optimizing and enhancing the material properties can improve the SFSA's performance; this finding serves as important technical information for users, for example, for high‐precision applications such as microsurgery, maintaining error rates below 5% requires using an SFSA‐based device within a 4‐day timeframe. Conversely, tasks demanding lower accuracy levels can tolerate usage periods.

#### 2.2.3 Finite Element (FE) Simulation

On top of the mathematical model, here we report the results of simplified simulations conducted using COMSOL Multiphysics 6.0.0.405, to provide a better understanding of the SFSA operation. The simulations involved two small segments of master and slave muscles with 10% of the actual muscles’ length to reduce computational complexity while still capturing essential aspects of the system's behavior. There are two phases in the simulation including elongating two muscles (or pressurizing) and applying displacement to the master muscles (or pulling) (Note S2, Supporting Information). Instead of using Fluid‐Structure Interaction (FSI), we employed a volumetric constraint method (VCM). This decision was made due to the complexity of FSI simulations and computational resources required for such analyses, while the VCM can still provide the approximately correct behavior of the SFSA with very low simulation time. Figure 2G plots the results after Phase 1 and Phase 2 of this simulation, while Figure 2H depicts the comparison of the simulation results with the mathematical and experimental data in terms of the muscles’ strain (Movie S1, Supporting Information). The FE simulation can closely predict the trends and relationships between the slave and master muscles. The slope of the FE simulation result is a bit higher than those of the mathematical and experimental results; this is because the muscles simulated here are much shorter than the physical prototype. Thus, future work should include an attempt using the same length as the physical prototype and considering the FSI simulation to closely predict the results of physical experiments.

#### 2.2.4 Tuning the Motion Sensitivity by Changing the Physical Configuration of the SFSA

As discussed above, the motion sensitivity can be tuned by changing the initial pressure; however, changing the initial pressure may change the workspace of the surgical tools and may not scale enough even at very high pressure. Thus, we propose an additional way of tuning the motion sensitivity by changing the configuration of the master muscles. Specifically, we can improve the motion sensitivity of the SFSA by meandering the master muscle. We validated this theory by experimenting with an SFSA frame consisting of an Ø2.5 × 200 mm master muscle and an Ø2.5 × 50 mm slave muscle. The master muscle was meandered with an increasing number of meandering turns from zero to three turns. Details of the experimental setup for this test can be found in Figure S6 (Supporting Information) (Note S3, Supporting Information) and Section 4. Upon examining each test, it is evident that there is an approximately linear relationship between the input and output motions. This can be explained by a relatively small displacement used in the experiments (2.5% of the original length of the master muscle) (Figure 2B (left)). As calculated, the motion sensitivity of the SFSA jumped from ≈0.38 (mm mm^−1^) to roughly over 1.15 (mm mm^−1^) when the number of turns increased from *n*  =  0 to *n*  =  3 turns (Figure 2B (right)). This result indicates that the motion sensitivity will be increased if we keep increasing the number of meandering turns, however, it will require higher input forces to activate. By contrast, we can also reduce the sensitivity of the SFSA by coiling the master muscle along an elastic tube (Figure S7, Supporting Information). Like meandering, we implemented the experiment using the same SFSA frame; and the motion sensitivity dropped from ≈0.37 to ≈0.17 (mm mm^−1^) when the number of coilings increased from *n* = 0 to *n* = 6 turns (Figure 2C). Compared to the performance of the meandered SFSA, the coiled SFSA has unprecedented hysteresis, which can be attributed to the addition of the elastic tube and the unwanted friction between the muscle and the tube. Nevertheless, this challenge could potentially be surmounted through improved architectural design and the use of advanced materials for the master muscle. As an example, transforming the coiled master muscle and its flexible tube substrate into a unified muscle composite could effectively eliminate the unanticipated friction between the muscle and the rubber tube.

#### 2.2.5 Force Characterization

To better understand the SFSA performance in terms of the axial force sensitivity, we also performed another experiment, which has set up details shown in Figure S8A (Supporting Information). We first examined the SFSA consisting of a master muscle (Ø2.5 × 50 mm) and a slave muscle (Ø2.5 × 50 mm) with five different initial pressures. Results in Figure 2D (left) suggest that an increase in initial pressure corresponds to heightened force sensitivity, mirroring the observed trend in motion sensitivity. In addition, at higher pressures, the relationship between the input and output forces presents a more pronounced linearity when compared to lower pressures. This can be explained by the fact that higher pressure results in a greater potential energy, requiring a comparatively lower input force to activate and attain a specific level of output force. Force sensitivities, changed by the initial pressure, were also calculated (Figure 2D (right)), showing that the force sensitivity increased threefold, reaching roughly 0.28 (N N^−1^), as the initial pressure increased from 0.28 to 0.92 (MPa). We have also demonstrated that force sensitivity can be enhanced by modifying the configuration of the slave site (Figure S8B, Supporting Information). In this test, a high initial pressure of 1.05 (MPa) and a long master muscle (ø2.5 × 200 mm) were employed, which can be ascribed to the increased linearity observed in the data presented in Figure 2E (left) as compared to Figure 2D (left). As can be seen clearly from Figure 2E (right), the force sensitivity rises from ≈0.34 to 0.73 (N N‐1), a 100% increase, when the number of slave muscles is increased from one to six muscles. With this almost linear trend, we can predict that if we further increase the number of slave muscles, the force sensitivity potentially reaches 1 (N N‐1). This would help provide haptic force feedback in TSRSs when we can convert the 1‐to‐1 force sensation.

#### 2.2.6 Sensing Ability

Through our research, we discovered that the SFSA can offer not only axial force sensing but also strain sensing capabilities. In the force characterization described earlier, the signals of internal pressure were also recorded and converted into pressure drops shown in Figure 2F (left). This revealed linear correlations between the applied forces and the changes in internal pressure. Furthermore, force sensing sensitivities were performed based on the data, showing that the SFSA has high sensitivity, with an increase from ≈0.042 to ≈0.08 MPa N^−1^ when the initial pressure increased from 0.28 to 0.92 MPa (Figure 2F (right)). This force‐sensing ability holds significant utility within the realm of the SRSs, particularly for quantifying the interactions between the slave robot and its working environment. The strain or motion sensing ability of the SFSA, on the other hand, can estimate the robot's state for the SRS based on its displacement. It is interesting to note that the behaviors of strain‐sensing sensitivity are distinct to the change of boundary conditions and the initial pressure. For example, in scenarios where the slave muscle end was unconfined, as demonstrated in the motion characterization test (Figure S5, Supporting Information), the result of the pressure drops derived from experimental data—presented in Figure S3E (Supporting Information)—reveals that the sensing sensitivities remained nearly unchanged at roughly 23 kPa mm^−1^, despite the elevation in initial pressure. By contrast, in the case that the slave muscle end was held, as illustrated in the force characterization test (Figure S8, Supporting Information), the motion sensing sensitivities increased as the initial pressure increased, as can be seen in Figure S3F (Supporting Information). This difference appears because of changes in boundary conditions; now, the slave is fixed axially, meaning it cannot contract as it did previously. In this case, when the master is forcibly extended, both the slave and the master tubes are forced to stretch radially inward to maintain the constant volumetric constraint. This radial inward stretch, or tube wall thickening, causes the de‐stressing of the initially pressurized tube, which in turn depressurizes the fluid within. Thus, changing the initial pressure will cause an alteration in the rate of change of pressure drop within the fluid (Figure S3F, Supporting Information).

### 2.3 Applications

#### 2.3.1 SFSA‐Driven Soft Robotic Catheter

**Figure 3:**
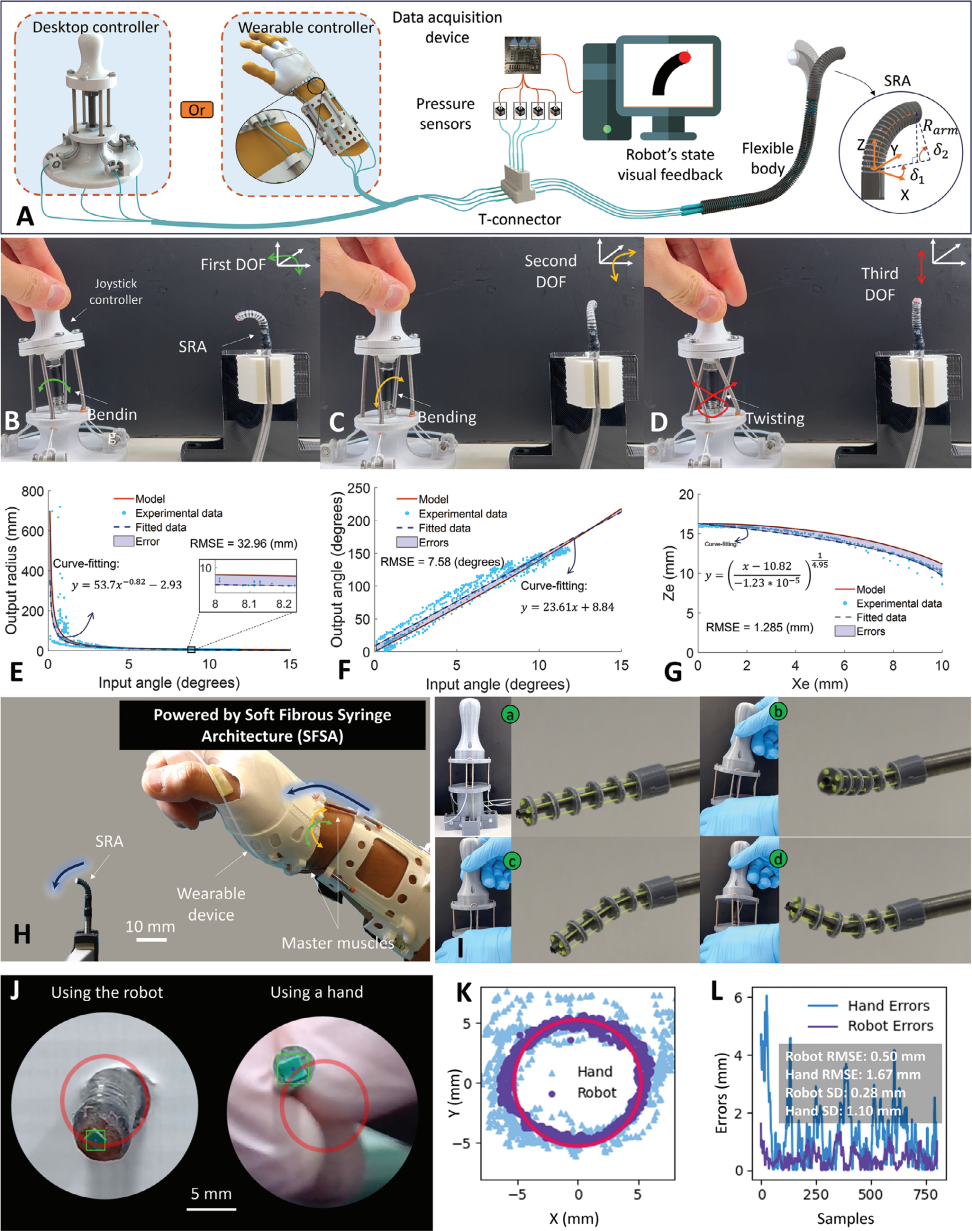
A) Overview of an omnidirectional soft robotic catheter system. Desktop and wearable controllers can directly control the soft robotic arm (SRA). Four pressure sensors are used to read the pressure changes in four SFSAs, providing sensing feedback to the users (Movie S2, Supporting Information). B–D) Demonstrations of the desktop device showing three DOFs (two bending and one translation) (Movie S3, Supporting Information). *Experimental data versus model of the desktop version for* E) bending radius, F) bending angle, and G) position of the end‐effector in the XZ plane. The model and data‐fitted curves are compared through Root Mean Square Errors (RMSEs). H) Demonstrations of motion control of the wearable device presenting two bending DOFs (Movie S4, Supporting Information). I) Demonstration of motion control of the SFSA‐cable continuum robot (Movie S5, Supporting Information). J) Demonstrations of reducing hand tremors (Movie S11, Supporting Information). K) Experimental data of the robotic catheter tip and the needle tip moved by hand following a defined circle (radius of 5 mm). L) Computed errors of the recorded positions compared to the defined curve.

So far, teleoperated soft robotic catheter systems have a wide range of applications across a spectrum of disciplines encompassing the medical realm (cardiac interventions, endoscopy, interventional radiology, etc.). In these diverse applications, their primary functions revolve around remote motion control for precise navigation and manipulation, coupled with their ability to perform state and environmental sensing. Typically, a soft catheter system consists of an omnidirectional soft robotic arm (SRA), which is meticulously governed by a master interface. In this research, we employed the SFSA for the development of a teleoperated soft robotic catheter system (**Figure** **3**A) to demonstrate its capabilities.

Our catheter system was built from four identical SFSAs including four slave muscles for the SRA and four master muscles for the user interface (see the third row in Table S1, Supporting Information). Each SFSA was integrated with an external pressure sensor via its fluid transmission tube to measure the change in inner pressure, which was then processed and sent back to the user (Figure 3A). Four slave muscles were assembled and covered by a heat shrink tube and a thin Ecoflex 00–30 (Smooth‐On Inc.) membrane to create the SRA, which is 12 mm in length and 3.4 mm in diameter (Figure S2A, Supporting Information). This structure offers bending compliance for the SRA. Once a difference in length between slave muscles appears, the SRA starts bending. By contrast, when all four slave muscles elongate or contract simultaneously, the SRA will be elongating or shortening, respectively.

Given the fibroid morphology of the four SFSA master muscles, the user interface can be designed in many ways. In this research, we established two types of user interface versions: desktop and wearable. Details of fabrication for user interfaces are shown in Note S1 (Supporting Information). Essentially, with the desktop version the user can rotate the joystick to change the length of the master muscles, which in turn affects the lengths of four slave muscles and, as a result, bends the SRA (Figure 3B,C). It is worth noting that twisting or pulling the joystick would simultaneously lengthen the stretched length of four master muscles, so that the SRA length would be shortened, giving the third DOF of the device as shown in Figure 3D and Movie S3 (Supporting Information). By performing this gesture, we can specifically change the workspace of the SRA given in situ conditions, which is one of the significant novelties of the SFSA‐based system. Despite advances, the use of the desktop version with rigid components may contradict the surgeon's operating habits and feelings during conventional surgery, where the surgeon directly operates a flexible catheter.^[^ ^3^ ^]^ The WUI version, on the other hand, is made of flexible materials, which may benefit the ergonomic user experience (Figure 3H). Although this device can only transfer two DOFs of the human wrist to control the bending motion of the SRA, this is the first time, a wearable device can directly convert the user's motions to control the surgical robotic instrument without using electrical motors and additional motion tracking sensors (Movie S4, Supporting Information).

During the design process, we developed an analytical model from the SFSA model for the desktop version to better understand the relationship between the input motion of the joystick and the output motion of the SRA. Briefly, the corresponding length of the *i^th^* slave muscle (*i*  =  1,  2,  3,  4) is obtained as follows:

(11) $$l_{si}=F_{SFSA}\left(P_{initial},l_{mi}\right)$$

where *F_SFSA_* is the function of the SFSA model with the inputs are the initial pressure *P_initial_* and the instantaneous lengths of the master muscles *l_mi_*, which are defined from the geometry of the desktop version (Figure S2B, Supporting Information). Details of the derivation have been presented in Note S2 (Supporting Information). We assume that the slave muscles of the SRA have negligible effects on each other when their lengths are changed. So the SRA's bending motions can be identified by three parameters (Figure 3A) including the bending radius *R_arm_* or curvature, the angle of the bending plane with respect to the *X*‐axis δ_1_, and the bending angle of the arc of the SRA δ_2_:^[^ ^35^ ^]^

(12) $$\left\{\begin{array}{c} \delta_{1}=sign\left(\Delta_{y}\right)\cos^{-1}\left(\frac{\Delta_{x}}{\Delta }\right)\\ \delta_{2}=\frac{\Delta }{R_{dt}}\\ R_{arm}=\frac{l_{s}}{\delta_{2}} \end{array}\right.$$

where $\Delta =\sqrt{\Delta_{x}^{2}+\Delta_{y}^{2}}=\sqrt{{\left(l_{s4}-l_{s2}\right)}^{2}+{\left(l_{s3}-l_{s1}\right)}^{2}}$ and $l_{s}=\frac{1}{4}\sum_{i=1}^{4}l_{si}$. It is noted that if we consider the wrist as a ball joint, the model for the wearable version would be similar to that of the desktop version. Based on the kinematic model of the SRA, we estimated its workspace (Figure S2C, Supporting Information), which is extendable and composed of a set of concentric spheres with increasing radius and partially missing bottom areas. Additionally, based on the SFRM model (*F_SFRM_*) we can also estimate the corresponding lengths of the slave muscles using the instantaneous internal pressures ($P_{internal\_i}$, i=1,2,3,4) measured by the pressure sensors as $l_{si}=F_{SSA}\left(P_{internal\_i}\right)$. Substituting this to Equation (12), we can also obtain the kinematic state of the SRA.

We experimented to validate the relationship between the input motion of the desktop version and the output motion of the SRA. As shown in Figure S9 (Supporting Information), the motion of the device is characterized in a 2D plane, where input and output motions are captured and processed by OpenCV. We performed curve‐fitting for each set of experimental data and compared the developed model curve with the fitted curve by using Root Mean Square Errors (RMSEs). According to the results depicted in Figure 3E–G, the RMSE for the radius is 32.96 mm, which is reasonable given that the SRA starts bending at extremely large radii (up to infinity), while the RMSEs for bending angle and end‐effector position are of ≈7.58 degrees or 0.13 rads and 1.285 mm, respectively, which are less than 8% compared to the testing ranges. These errors can be ascribed to a multitude of factors, where non‐uniform fabrication of muscles and the lack of absolute symmetry in the assembly of the system leading to out‐of‐plane bending motions are the main contributors. Despite that, in terms of trend, there are notable concordances observed between the experimental data and the model of the instantaneous bending radius, bending angle, and the position of the end‐effector, respectively. Further, we employed the model and the internal pressure measured by the pressure sensors to provide real‐time visual feedback of the SRA's motion, which would be highly beneficial for users during the navigation process (Figure S2D; Movie S2, Supporting Information). This demonstration serves as compelling evidence of the SFSA's novel capabilities when delivering motion control and robot‐state sensing without using electrical motors or additional electronics at the end‐effector, which have been observed in other soft robotic technologies.

#### 2.3.2 Motorless Control of Cable‐Driven Flexible Robot

Currently, the control of a cable‐involved continuum robot featuring multiple DOFs necessitates the utilization of electrical motors in conjunction with a gear and pulley mechanism. Additionally, the ratio of the input and output motions is typically confined, requiring a comprehensive system overhaul to modify it. Thus, this paper proposes a novel approach involving the integration of an SFSA with cables to control the multi‐DOF cable‐driven continuum robot. Figure S2E (Supporting Information) presents the overview of the system, where a cable‐driven continuum robot is controlled by an SFSA‐based joystick controller. Like the soft robotic catheter system, four SFSAs were employed with the master muscles assembled within the controller. The slave muscles, on the other hand, were integrated into a slave tuning mechanism and linked with four cables of the continuum robot. Details of the controller and the slave tuning mechanism can be found in Figure S2E,F (Supporting Information). Thanks to the use of SFSAs, the input–output motion ratio can be tuned in situ while not requiring the use of any electrical motors and complicated control systems. Figure 3I showcases the demonstrations of motion control of this system with several gestures. Since backlash and hysteresis remain, it is necessary to develop the SFSA design. This will help move the conjunction between the SFSA slave muscles and cables closer and closer to the continuum robot's end‐effector without increasing its body size.

#### 2.3.3 Tremor Reduction

In effect, hand tremors have negative impacts on the control accuracy of surgical FRSs, especially in microsurgery.^[^ ^36^ ^]^ Given the adaptable (tunable) MSF (i.e., the ratio of output/input motion) of the SFSA, the proposed robotic catheter can be able to reduce the hand tremors effectively and allow precise motion compared to other systems (e.g., the ones that have an MSF = 1 or a fixed MSF) and manual operation. To demonstrate this ability, we have experimented by recording the robotic catheter tip controlled by the SFSA master controller and the needle tip moved by hand (representing a system that has MSF = 1) (Figure 3J; Movie S11, Supporting Information), when they were tracking a defined trajectory—a 5 mm‐radius circle. The results (Figure 3K) reveal that by using our system, the errors have been significantly reduced by about over three times with the computed RMSE decreased from 1.67 to 0.5 and SD dropped from 1.1 to 0.28 (Figure 3L). The efficiency of the SFSA‐based catheter would be even higher when it comes to the smaller sizes or the more of the trajectory.^[^ ^11^, ^36^, ^37^ ^]^

#### 2.3.4 SFSA Sensing Applications for a Soft Robotic Catheter

**Figure 4:**
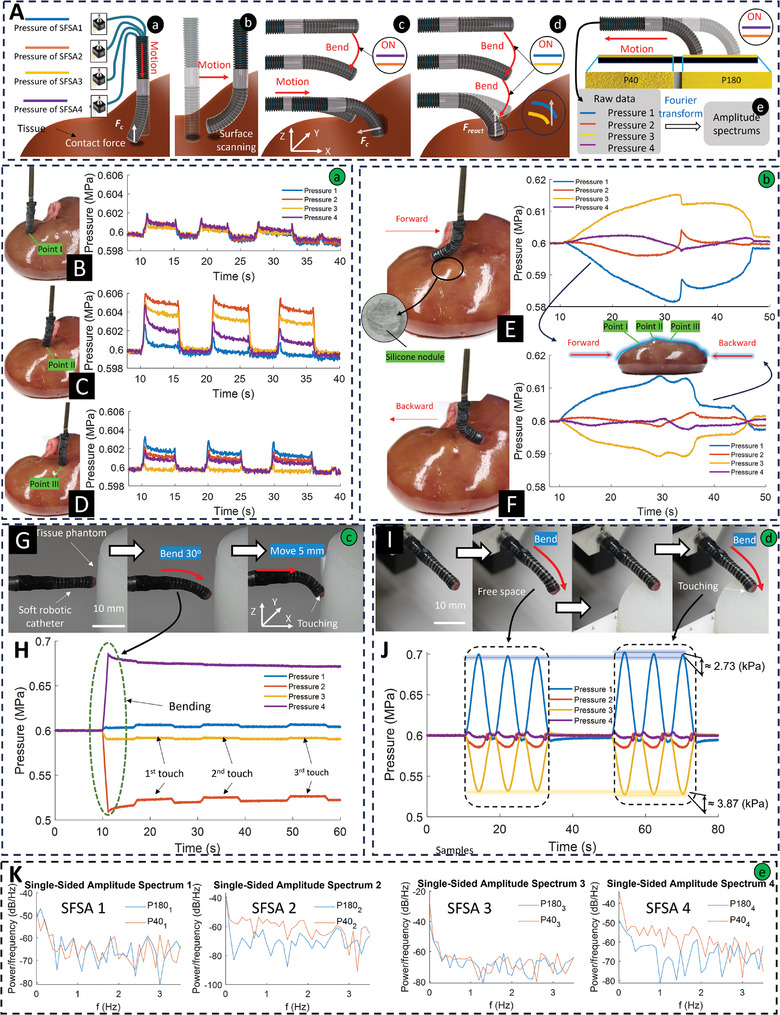
Demonstrations of the SFSA sensing capabilities within the soft robotic catheter: A) Experimental setups for (a) passive sensing with equal initial pressures for stiffness detection; (b) surface scanning/mapping; (c) passive touch sensing with different initial pressures; (d) active touch sensing; (e) texture detection with a data processing method. S*tiffness dete*ction (Movie S6, Supporting Information) B) The palpation on Point I of normal tissue. C) The palpation on Point II of a simulated tumor (which has a silicon nodule underneath). D) The palpation on Point III of the simulated tumor margin. S*urface mapping* (Movie S7, Supporting Information) E) Surface mapping of the kidney surface when moving the catheter in a forward direction. F) Surface mapping of the kidney surface when moving the catheter in a backward direction. *Passive touch sensing* G) The soft catheter is bent and moved to touch the phantom along the *X*‐axis (Movie S8, Supporting Information). H) The measured pressure signals over the testing period. *Active touch sensing* I) The soft catheter is bent in free space and touches with the tissue phantom. J) The measured pressure signals over the testing period with computed disparities between bending in free space and actively touching (Movie S9, Supporting Information). *Results of texture detection* K) Single‐sided amplitude spectrum of Fast Fourier Transform (FFT) analysis of four SFSAs’ internal pressure data.

In this section, we report our investigation into the built‐in sensing abilities of the SFSA applying for the soft catheter to sense the interactions with the surrounding environment without requiring additional onboard sensors. This research has four main applications: stiffness detection or palpation, surface mapping/scanning, passive/active touch sensing, and texture detection (**Figure** **4**). To obtain the quantitative estimation of each sensing application, we have performed characterizations for both passive (Figure S10, Supporting Information) and active (Figure S12, Supporting Information) sensing sensitivities with details provided in Note S4 (Supporting Information). While the force data can be roughly converted using established characterizations, we opted to directly present pressure data to clearly and precisely represent the soft catheter's properties.

First, the sensitivities of four SFSAs are equal to ≈0.025 MPa N^−1^, when the catheter has a straight shape with equal initial pressure (0.6 MPa) in its four SFSAs (Figure S10A,B, Supporting Information). At this stage, the soft straight catheter could be used for stiffness detection/palpation and surface mapping. To justify, the catheter was moved along the *Z*‐axis (Figure 4Aa) to establish contact with a lamb kidney at three variant points (forth and back 3 mm three times) (Movie S6, Supporting Information): Point I—normal tissue (Figure 4B); Point II—a silicone nodule (Solaris, Smooth–On Inc., USA) (Figure 4C); and Point III located at the nodule margin (Figure 4D). As a result, the pressures in four SFSAs at Point I increased slightly by the same amount of ≈0.001 MPa. By contrast, at Point II, the pressure increments in four SFSAs are clearly distinct and significantly higher than those at Point I, where the measured pressure in SFSA 2 presents the greatest change (0.005 MPa), roughly 10 times higher than that of SFSA 1. This happened because the tissue stiffness was higher which increased the contacting force, inducing deformation of the catheter. Although the variations at Point III are less significant than those at Point II, they are still discernible. Furthermore, we examined the catheter's surface mapping capability (Figure 4Ab) by sliding it forward and backward on the surface of the lamb kidney (Figure 4E,F). The outcomes of this experiment validate the catheter's proficiency in surface mapping as the recorded pressures in SFSAs 3 and 1—the most impacted—present the form of the kidney surface (Movie S7, Supporting Information). Notably, quick and substantial pressure changes were noticed when the catheter reached Point II, which matched the position of the previously created silicone nodule. This abrupt change in pressure indicated the catheter's ability to detect specific tissue defects. Similarly, at Point III, which represented the simulated tumor's edge, the pressure variations revealed a comparable abrupt shift, emphasizing the catheter's ability to pinpoint transitions between various tissue sections. The dramatic pressure differences shown at Points II and III highlight the catheter's accuracy in surface mapping, as it correctly identified areas of interest. These results of the passive sensing capabilities of the straight soft catheter bode well for medical diagnostics, where it might aid in detecting and localizing anomalies within tissues during minimally invasive operations.

Second, the passive sensing sensitivities of the catheter with different initial pressures applied to four SFSAs were characterized when it bent at 15, 30, and 45 degrees (Figure S10C–H, Supporting Information). By contrast to the straight soft probe, the bending probe exhibited disparities in sensitivities of four SFSAs. The differences were more obvious when the bending angle increased and the SFSA with the lowest initial pressure presented the highest sensitivity (e.g., SFSA 3 sensitivity was 0.14 MPa N^−1^ at 45 degrees Figure S10H, Supporting Information). We conducted the demonstration for this by moving the 30°‐bending catheter toward a soft tissue phantom (Ecoflex 00–30) (Figure 4Ac,G) to establish three touches. Figure 4H shows clear pressure changes in SFSA 2—which had the lowest initial pressure—representing the three touches. This occurred given that the SFSA with the lowest initial pressure (i.e., the shortest length as the catheter bends toward it) experiences the greatest reaction force due to the probe's movement direction being parallel to its body and perpendicular to the tissue surface. While palpation typically involves a probe movement perpendicular to the tissue surface, alternative probe orientations can be employed. Figure S11 (Supporting Information) illustrates a scenario where the catheter was moved in a different direction, resulting in pressure changes among the SFSAs that deviated from the typical pattern, with the SFSA exhibiting the highest initial pressure undergoing the most significant pressure change. This opens a great potential ability to determine the force direction, which will be explored in future studies. Additionally, the bending catheter also demonstrated surface scanning capabilities, as evidenced by the captured profiles of a circle and a spiked triangle in Figure S13 (Supporting Information).

Third, we explored the active touch sensing capability of the soft robotic catheter with experimental characterization detailed in Figure S12 of Note S4 (Supporting Information). As a result, two pairs of the SFSAs were observed to have opposing pressure trends, following their established patterns of either increasing or decreasing pressure. This has been demonstrated by the experiment presented in Figure 4I,J, where the pressure of the SFSA 3 further dropped by ≈3.87 kPa (decreased in free space), while that of SFSA 1 raised by 2.73 kPa (increased in free space) at the maximum input motions (Movie S9, Supporting Information). The observed disparity can be explained simply by the reaction force from the tissue phantom acting significantly on the slave muscles of SFSAs 1 and 3. This force‐imposed constraint on SFSA 3, inhibited its intended shortening of the slave muscle, thereby reducing stress and causing a subsequent drop in inner pressure. In contrast, the force constrained SFSA 1 from carrying out its intended lengthening of the slave muscle, resulting in increased stress and a subsequent rise in inner pressure.

Finally, we investigated the texture‐detecting ability of this soft robotic catheter by using two distinct texture samples of P40 and P180 sandpapers (Figure 4Ae), on which the catheter was sliding (Figure S14, Supporting Information). The obtained pressure signals were then subjected to Fast Fourier Transform (FFT) analysis (Figure 4Ae). Here, we consider a low‐frequency range from 0 to 3 Hz of FFT for the signals of these two texture samples. As a result, the difference is more obvious in the signals of SFSAs 2 and 4 since they were more influenced (Figure 4K). Specifically, the P40 sample (the rougher) exhibits prominent peaks compared to the P180 sample (the finer) in the single‐sided amplitude spectrum of these SFSAs. These findings indicate that the catheter effectively differentiated between the two samples based on their texture‐related pressure responses.

## 3 Discussion and Conclusion

In this study, we introduce a new architecture, the SFSA, which presents a novel approach to electricity‐free actuation and motorless control for FRSs. It overcomes the limitations of existing actuator methods with rigid electric pumps/pistons/DC motors, which are mostly employed in soft hydraulic actuators. These methods are inherently associated with bulky, high‐cost, and complex systems, leading to the complexity of controlling FRSs (i.e., due to the inherent properties of soft materials and nonlinear behavior).^[^ ^1b^ ^]^ Our SFSA is also electricity‐free and possesses a high working pressure range compared to soft electric‐driven pumps,^[^ ^19b,c^ ^]^ and exhibits faster response and higher versatility due to its fibroid structures than compressible‐fluid‐based methods.^[^ ^21^ ^]^ Additionally, SFSA features multimodal, built‐in sensing capabilities for FRSs, unlike previous studies that typically necessitated supplementary components (e.g., optical sensors^[^ ^38^ ^]^ and embedded materials)^[^ ^24^, ^39^ ^]^ or additional fluid channels^[^ ^26^ ^]^ for their end‐effectors to include sensory capabilities. SFSA can be fabricated from commercially available, cost‐effective components by simple means (Note S1, Supporting Information).

The fundamental SFSA was demonstrated to be capable of providing mostly linear, low hysteresis relationships between input and output motions and forces over a designed range of displacements (Figure 2E; Figure S3B, Supporting Information). The properties of the SFSA provide tunable motion, sensing, and force sensitivities for specific applications through different ways such as modifying inner pressure or configuration, that were characterized in this research. Changing the SFSA configuration (e.g., meandering and coiling the master muscle) could be achieved during the design phase to meet specific needs. On the other hand, adjusting the SFSA internal pressure is particularly advantageous when dealing with specific applications or in situ where technical constraints (e.g., limited size and motion capabilities of the robotic catheters)^[^ ^10^, ^40^ ^]^ are strictly required. On top of that, altering the material properties and manufacturing methods can also adjust the SFSA sensitivities. Accordingly, we provided two methodologies encompassing a mathematical model and an FE model to predict these technical modifications. The preliminary data gathered from this research affirms the efficacy of these modeling approaches. Nonetheless, future investigations employing a dynamic mathematical model^[^ ^41^ ^]^ and FSI simulation^[^ ^42^ ^]^ are still necessary to improve the predictive capabilities concerning the interactions of the device with the surrounding environment within its working space.

A soft robotic catheter powered by the fundamental SFSA was developed in this study to justify SFSA's capabilities of motor‐free controlling and built‐in sensing. Specifically, the catheter's motion was demonstrated to be motor‐free‐driven by two different versions of controllers, including the desktop version and the WUI (Figure 3A). While the desktop version can provide 3‐DOFs for controlling the catheter's motion, the use of rigid components and position‐fixed requirements make it less ergonomic and comfortable than the WUI.^[^ ^3^ ^]^ Through this step, we further applied the SFSA mathematical model for the catheter system and validated it, resulting in acceptable errors compared to the experimental data. This confirms that designers can rely on the mathematical model to anticipate the workspace, and input and output motions when developing SFSA‐based systems. Additionally, the MFS of the SFSA‐based catheter effectively mitigates hand tremors, as demonstrated by improved motion control compared to manual operation and systems with an MFS of 1. This key factor of the SFSA significantly facilitates the application of the soft robotic system in micro‐scale tasks.^[^ ^11^, ^36^, ^37^ ^]^

When it comes to the built‐in sensing capabilities of the SFSA, we demonstrated that the SFSA‐based catheter's motions can be regenerated as visual feedback (Figure S2D, Movie S2, Supporting Information) thanks to the pressure signals and the developed mathematical model. This information can greatly support the surgeons during surgical procedures (e.g., navigation of the catheter in the blood vessels).^[^ ^43^ ^]^ The catheter can also recognize touching events between its end‐effector and its surroundings while either passively or actively controlled. In addition, the SFSA's abilities of texture and stiffness detection, as well as surface mapping were also examined (Figure 4). While texture detection requires additional FFT analysis to be recognizable, the results of stiffness detection and surface mapping are obvious. It is worth highlighting that these sensing capabilities of the SFSA do not require any add‐on electrical elements or channels for the end‐effectors, but they only need a commercially available pressure sensor for each SFSA, which can be located outside the operational site. Additionally, the sensing resolution of SFSA can be simply tuned (in situ) by changing the initial pressure of the system. This addresses the constraints observed in prior research reports, where the necessity for redesigns or adjustments to component sizes^[^ ^27^ ^]^ was evident. These abilities hold significant potential across various applications, such as locating the position of tumors and identifying their stiffness in medical diagnosis procedures. Despite advances, the output of the SFSA's sensing within the soft catheter is currently limited to pressure‐based signals. This signal type demonstrates clear potential to be converted into reaction forces.^[^ ^44^ ^]^ Future endeavors could focus on refining calibration techniques for specific contacting areas and the development of a robust mathematical model to enhance the versatility and accuracy of SFSA in medical applications.

It is worth noting that the SFSA applications are not only limited to this fundamental frame but also employed by other teleoperated systems using distinct structures, sizes, and artificial muscle types for different purposes (Figure S1A, Movie S10, Supporting Information). For example, a submillimeter (Ø0.8 mm) slave SFRM can be controlled by a master (Ø2.5 mm) SFRM (Figure S1B, Supporting Information), holding potential for developing submillimeter catheters or medical tools accessing small vessels and chambers of the human body. Smart textiles, created from weaving a long (up to 1000 mm) SFRM with yarns/fabrics, can apply SFSA to drive a soft robotic arm with a 3D‐printed outer sheath (Figure S1C, Supporting Information).^[^ ^40^ ^]^ These smart textiles can also control a twisted SFRM for lifting weights or teleoperating a robotic skeleton arm (Figure S1D, Supporting Information) and a circular soft fluidic fabric muscle sheet (FFMS) (Figure S1E, Supporting Information).^[^ ^45^ ^]^

However, several areas of the SFSA can still benefit from additional development, such as the emergence of hysteresis at large working displacements, which potentially cause system delays. Additionally, the tunable sensitivities exhibit trade‐offs, given the fact that they are interrelated and dependent on various technical aspects. For example, if we increase the motion sensitivity through an increase in initial pressure, this also extends the end‐effector workspace, which may not be desirable. On the other hand, if we increase that motion sensitivity by meandering the master muscles, it will necessitate a higher activation force. However, these limitations can be effectively mitigated through mechanical design, optimization, and material selection. For instance, sensible material selection would greatly reduce the impact of hysteresis. Moreover, the relationships between these sensitivities and other mechanical parameters can be systematically investigated and modeled, facilitating purpose‐driven optimization when employing this technology.

In addition, environmental conditions can influence the SFSA performance. For instance, due to hydraulic fluid expansion and contraction, extreme temperatures (above 100 °C or below 4 °C) may compromise control accuracy or system function. However, the system could operate safely and reliably within typical medical temperature environments. In high‐precision application such as microsurgery, rigorous experimentation is critical to accurately characterize the influence of small temperature variations on device performance metrics, including linearity, hysteresis, and sensitivity. At the same time, dynamic environments with flow conditions can induce errors in the position control of the soft robotic catheter. In our dynamic test, detailed in Note S5; Figure S15 (Supporting Information), a maximum position error of 5% within the motion range was recorded, which was caused by an increasing input flow from 0 to ≈5 L min^−1^ (Movie S12, Supporting Information). While this error can be minimized by exploring compensation strategies, for example increasing the device's stiffness, more extensive dynamic tests are needed to be involved in future work to better understand the influence of the external environments.

In conclusion, the SFSA approach holds great potential in the development of motor‐free and electronics‐mitigated FRSs, with applications spanning from the medical field to industrial domains. Realizing the potential of such design hinges upon thorough research and strategic implementation of this technology.

## 4 Experimental Section

### SFSA's Motion Validation, Durability, and Response Latency Test

One end of the SFSA slave muscle (the first row in Table S1, Supporting Information) was connected to a free‐twisting mechanism and free to move longitudinally, while the other end was fixed to a 3D‐printed sliding tray. A laser sensor (Keyence, model IL‐100, Keyence Corp., USA) was used to measure the elongation of the slave muscle, which was set up to generate a laser line pointing perpendicularly to the marker mounted on the free‐twisting mechanism. Meanwhile, the master muscle was connected to and driven by a linear active motor (Zaber X‐LRQ‐E Series, Zaber Technologies Inc., Canada). A commercial pressure sensor (Honeywell, USA) was used to measure the instantaneous inner pressure of the SFSA system, which was decoded by a QPIDe Data Acquisition Device (QUANSER, Canada) and MATLAB Simulink (Mathworks, Inc., USA). Initial pressure was supplied by a 1 mL syringe and then held by a pressure lock after the SFSA inner pressure reached the target initial pressure (Figure S5, mSupporting Information). For the motion characterization test (Figure 2A), four distinct initial pressures of 1.05, 0.87, 0.68, and 0.46 MPa were introduced into the SFSA. At each initial pressure, the master muscle was pulled by the linear motor to remove any slack along its length. From this state, the master muscle was then pulled by the motor in a sinusoidal motion with a peak‐to‐peak amplitude of 20 mm and a frequency of 0.2 Hz, while the pressure and laser signals were recorded. Similarly, the durability and response latency tests, which have the results shown in Figure S3C,D (Supporting Information), were conducted based on this setup. Specifically, for the durability test the master muscle was pulled in a sinusoidal motion with a peak‐to‐peak amplitude of 2.5 mm and a frequency of 0.2 Hz in 1.5 h for seven consecutive days. For the response latency test, the sinusoidal motion included a peak‐to‐peak amplitude of 1 mm and a frequency of 0.2 Hz in 10 cycles.

### SFSA's Motion Sensitivity Tuning

For optimizing the SFSA's motion sensitivity, the same setup as the above section was used with the changes of the master muscle in length and configuration. To increase the motion sensitivity, a master muscle with a length of 200 mm (the second row in Table S1, Supporting Information) was used and meandered with an increasing number of turns from *n* = 0 to *n* = 3 (Figure S6, Supporting Information). The meandered master muscle was pulled by the motor in a sinusoidal motion with a peak‐to‐peak amplitude of 2.5 mm and a frequency of 0.2 Hz. Similarly, to reduce the motion sensitivity, the master muscle was coiled along an Ø3 × 120 mm natural rubber tube (MECCANIXITY, Dragonmarts Co. Ltd., Hong Kong) with an increasing number of turns from *n* = 0 to *n* = 6 and was pulled by the motor in a sinusoidal motion with a peak‐to‐peak amplitude of 5 mm and a frequency of 0.2 Hz (Figure S6, Supporting Information).

### SFSA's Force Characterization

For force characterization, a fundamental SFSA (the first row in Table S1, Supporting Information) was first fabricated. The single master muscle (Ø2.5 × 50 mm) was attached to a force gauge (Mark‐10, USA), which was assembled into the linear active motor, for measuring the input force. Meanwhile, the slave muscle (Ø2.5 × 50 mm) was linked to a load cell—LSB200 (FUTEK, USA) for recording the output force (Figure S8A, Supporting Information). The other components including the pressure sensor, pressure lock, and initial pressure supplier were set up similarly to Note S3 (Supporting Information). In this test, five distinct initial pressures of 0.28, 0.4, 0.5, 0.7, and 0.92 MPa were introduced into the SFSA (Figure 2D). The master muscle was pulled by the motor in a sinusoidal motion with a peak‐to‐peak amplitude of 12 mm and a frequency of 0.2 Hz, while the pressure, force gauge, and load cell signals were recorded. For evaluating the force sensitivity when increasing the number of slave muscles (from *n* = 1 to *n* = 6), a single muscle (Ø2.5 × 50 mm) was then added to the slave side after each test was finished (Figure S8B, Supporting Information). It is noted that a long master muscle (Ø2.5 × 50 mm) and a high initial pressure (1.05 MPa) were employed. To reach 4 N input force, the peak‐to‐peak amplitude of the input motion changed through each test. Specifically, it is 6 mm when *n* = 1, 9 mm when *n* = 2, 10.5 mm when *n* = 4, and 12 mm when *n* = 6.

### Motion Validation of The Soft Robotic Catheter

As the measurement of the input and output motions were performed digitally using computer imaging techniques with OpenCV, the SRA and joystick controller were set up in front of a green background to distinguish the color contrast (Figure S9, Supporting Information). Their motions were captured by a DSLR camera (NIKON D7500, Japan), which was directly facing the plane (*XZ* plane) where the objects were moving on. For the input angle, two rectangular makers were stuck on the joystick controller, where their central points (top and bottom points) were processed and used to calculate the input bending angle. The input angle is the angle between the *Z*‐axis and a line from the top point to the bending point, which is defined to be 10 mm away from the bottom point vertically. Meanwhile, a red marker was mounted on the end tip of the SRA, which was then used to calculate the output bending angle from the coordinate origin point (Figure S9, Supporting Information). Finally, the centerline of the SRA was processed based on the black color of the body, which then was used to estimate the output bending radius.

## Conflict of Interest

The authors declare no conflict of interest.

## Supporting information

Supporting Information

Supplemental Movie 1

Supplemental Movie 2

Supplemental Movie 3

Supplemental Movie 4

Supplemental Movie 5

Supplemental Movie 6

Supplemental Movie 7

Supplemental Movie 8

Supplemental Movie 9

Supplemental Movie 10

Supplemental Movie 11

Supplemental Movie 12

## Data Availability

The data that support the findings of this study are available from the corresponding author upon reasonable request.
