# Supplementary material for: Soft Fibrous Syringe Architecture for Electricity‐Free and Motorless Control of Flexible Robotic Systems
Source: Adv Sci (Weinh). 2024 Aug 19;11(39):2405610. doi: 10.1002/advs.202405610 (PMC11497036; doi:10.1002/advs.202405610)
Supplement: Supplementary file 1 — Supporting Information [file ADVS-11-2405610-s013.docx]

Supporting Information

Soft Fibrous Syringe Architecture for Electricity-Free and Motorless Control of Flexible Robotic Systems

*Chi Cong Nguyen, Trung Thien Hoang, James Davies, Phuoc Thien Phan, Mai Thanh Thai, Emanuele Nicotra, Amr Al Abed, Hien A. Tran, Thanh An Truong, Bibhu Sharma, Adrienne Ji, Kefan Zhu, Chun Hui Wang, Hoang-Phuong Phan, Nigel Hamilton Lovell, and Thanh Nho Do^*^*

*C.C.Nguyen, T.T.Hoang, J.Davies, P.T. Phan, M.T.Thai, E.Nicotra, A.A.Abed, H.A.Tran, B.Sharma, A.Ji, K.Zhu, N.H.Lovell, T.N. Do*

Graduate School of Biomedical Engineering, Faculty of Engineering, and Tyree Institute of Health Engineering (IHealthE), UNSW Sydney, Kensington Campus, Sydney, NSW 2052, Australia

*T.A. Truong, C.H.Wang, H.P.Phan*

School of Mechanical and Manufacturing Engineering, Faculty of Engineering, UNSW Sydney, Kensington Campus, Sydney, NSW 2052, Australia

*M.T. Thai*

College of Engineering and Computer Science, VinUniversity, Hanoi, Vietnam

*** Corresponding author**: Thanh Nho Do

**Email:**  [*tn.do@unsw.edu.au*](mailto:tn.do@unsw.edu.au)


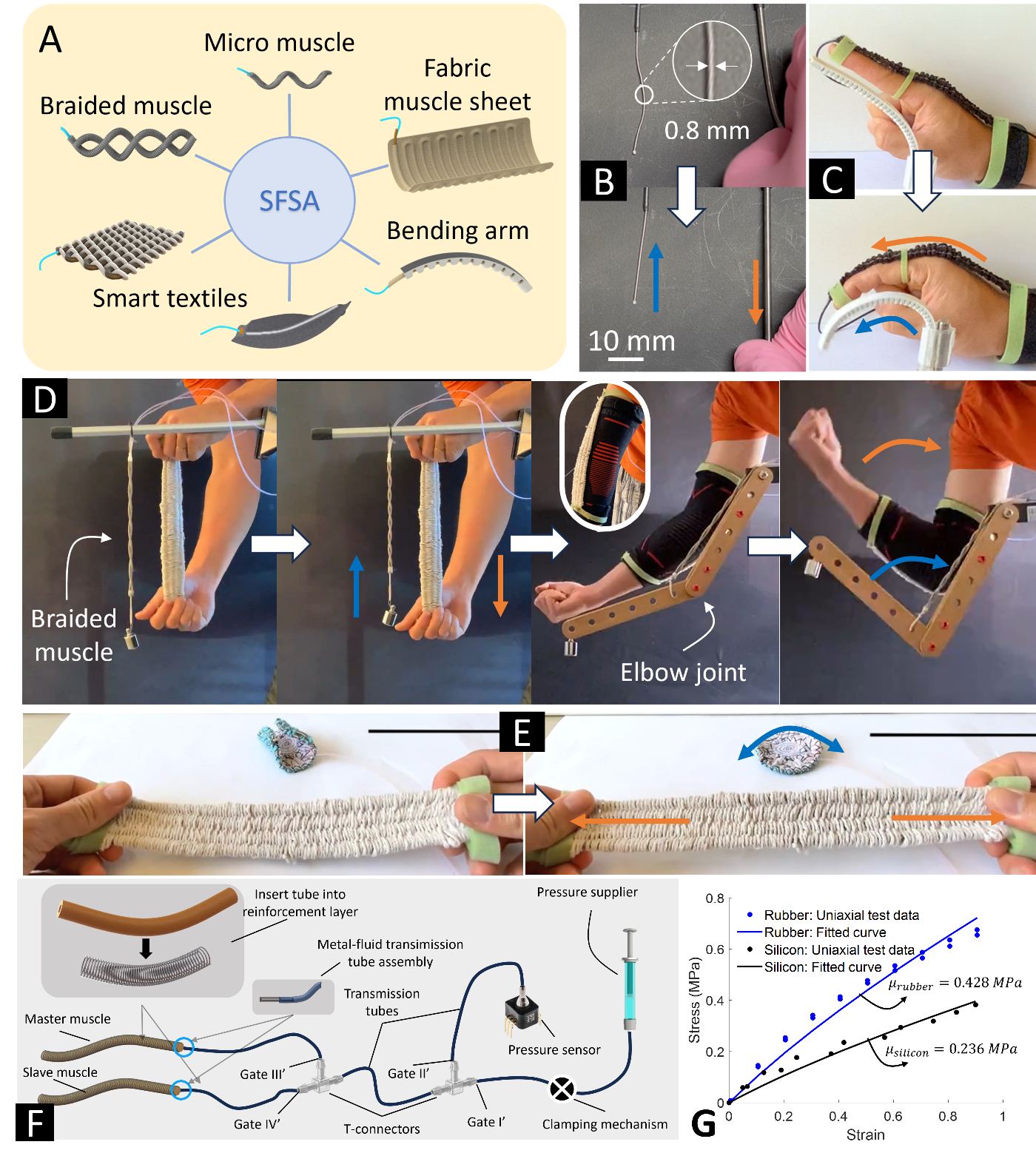


**Figure S1.** (A) Potential applications of SFSA to different types of soft hydraulic artificial muscles/actuators (**Movie S10**). (B) A SFRM is used to control a submillimeter SFRM. *Smart textiles formed from a long muscle and yarns/fabrics are used to control*: (C) a SRA with a 3D-printed outer sheath,^[1]^ (D) a twisted SFRM, and (E) a circular soft fluidic fabric muscle sheet (FFMS).^[2]^ (F) Fabrication methods of the fundamental SFSA. (G) Experimental stress-strain data and Neo-Hookean model-fitted curves for hyperelastic materials of the artificial muscles under uniaxial tensile loading.

# **Supplementary Note 1. Materials and Fabrication Methods**

In this section, we report the fabrication method of the fundamental SFSA and its application device - the soft robotic catheter systems. For the SFSA, we used three types of frames with different sizes of the muscles’ components (**Table S1**), however, their fabrication methods are similar. First, two soft, fiber-reinforced muscles (SFRMs) were made by inserting a rubber tube into an outer fiber reinforcement layer (**Figure S1F**). In this paper, we used two types of hyperelastic tubing; one is natural latex rubber tubing (MECCANIXITY, Dragonmarts Co. Ltd., Hong Kong) for the SFSA fundamental frame and the master muscles, and the other one is medical silicone tubing (Saint-Gobain S.A. Courbevoie, France) for the slave muscles of the soft robotic catheter. These tubing materials were characterized under uniaxial tension using a single-axis Instron (model 5543, Instron, USA) with the results shown in **Figure S1G**. Meanwhile, we used a stainless steel helical coil (Asahi Intecc Co. Ltd., Japan) for the fiber-reinforcement layer. Second, hollow metal tubes are then inserted into fluid transmission tubes (ColeParmer, USA) and glued in place (LOCTITE®, USA). Five metal-fluid transmission tube assemblies are then installed into a T-connector (McMaster-Carr, USA), creating a fluid duct system with four gates (**Figure S1F**): Gate I’ is for the hydraulic supplier; Gate II’ is for an external pressure sensor (40PC Series, Honeywell, USA); Gates III’ and IV’ are for the master and slave muscles, respectively. Fishing line knots are then tied at the joint locations, forming tight hydraulic seals, and are then glued in place. Then water is introduced through the transmission tubes and muscles before sealing the other ends of the muscles, while the sensor body is filled with water before being installed in Gate II’. After applying desired pressures, the pressure supplier is locked by a watertight clamping mechanism, then the SFSA is ready to be used.


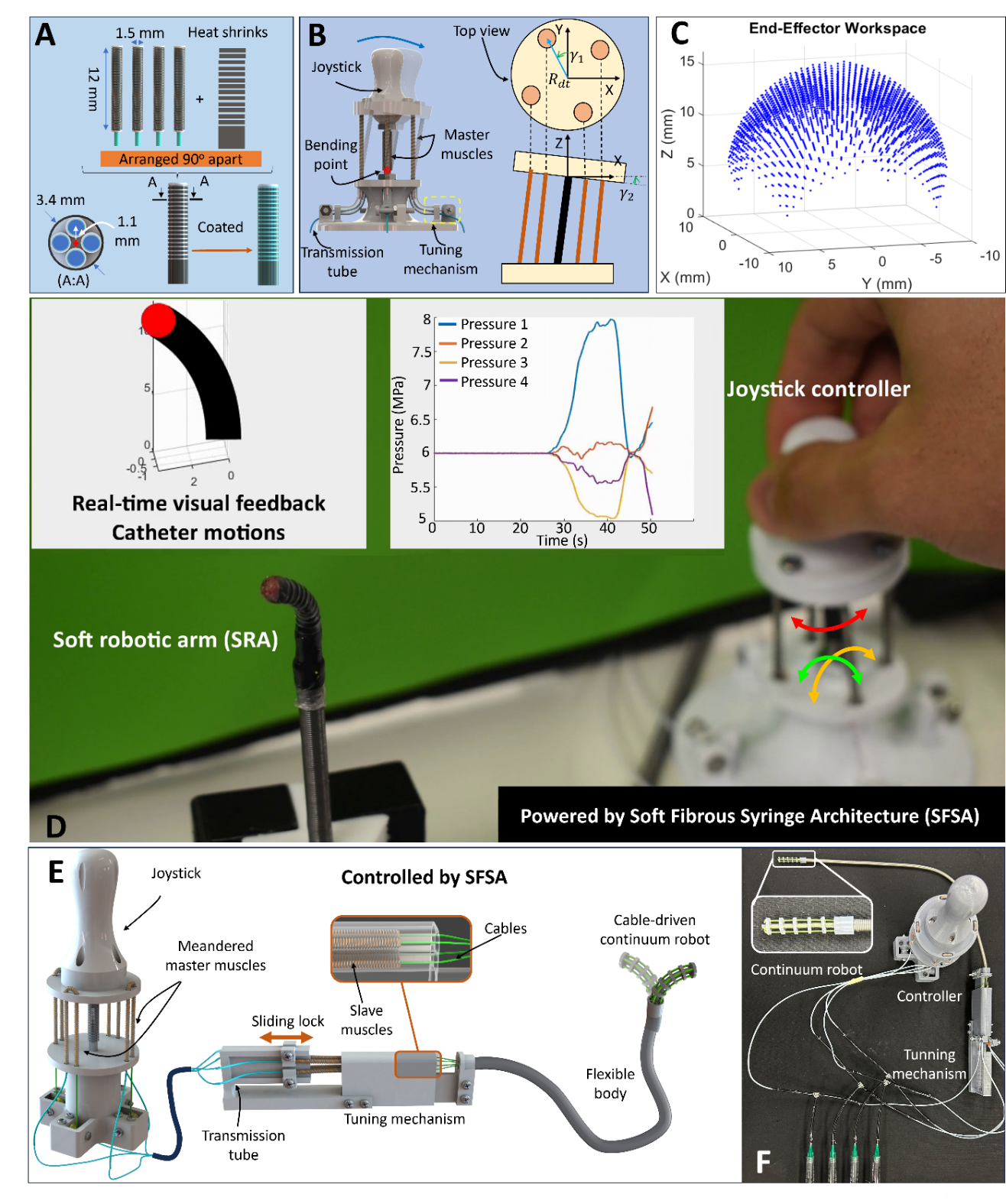


**Figure S2.** (A) Fabrication process and model of the soft robotic arm (SRA). There are two main steps in making the SRA including assembling four slave muscles and coating it with a thin layer of Ecoflex 00-03.^[3]^ (B) Analytical model of the desktop version, where the length of each master muscle depends on the azimuth angle $\gamma_{1}$ of the plate and $\gamma_{2}$ the tilted angle $\gamma_{2}$ of the plate. (C) Prediction of the end-effector’s workspace of the SRA in 3D Cartesian coordinate. (D) Demonstrations of motion control of the desktop device with real-time visual feedback of the soft robotic catheter (**Movie S2**). (E) Overview of a cable-driven continuum robot controlled by an SFSA-based controller. (F) The prototype of the SFSA-cable continuum robot.

**Figure 3A** (Main Manuscript) shows the overview of the soft robotic catheter system including the soft robotic arm (SRA) and the desktop and wearable controllers. For this system, we used four SFSAs with specifications shown in the third row of **Table S1**. Specifically, for the SRA, four slave muscles were arranged 90^o^ apart to function together and covered by heat shrinks (**Figure S2A**). After heating the heat shrinks, the SRA was super-glued at its end tip and coated with Ecoflex 00-30 (Smooth-on Inc., PA, USA). The desktop controller was created as a joystick device, where four master muscles had been lined up in a circle around the device’s central shaft. This central shaft was comprised of a sturdy spring, which allowed the joystick to be bent, twisted, and stretched (**Figure S2B**). There was a tuning mechanism, consisting of a screw, two bolts, and a string, attached to one end of each master muscle, which was responsible for calibrating and modifying the scaling factor for the system. Based on the desktop device, the initial stretched length of the muscle can be calibrated, making the alignment between the joystick and the SRA during the assembly process. Additionally, the motion sensitivity and workspace of the SRA can be varied even in in situ conditions based on this mechanism. The other parts of this device such as the joystick and base are designed and fabricated using an FDM 3D printer (Ultimaker, Netherlands) with PLA materials.

**Supplementary Table S1**. **Specifications of three different SFSAs**

| **No.** | **Master muscles** | | | **Slave muscles** | | | **Purpose** |
| --- | --- | --- | --- | --- | --- | --- | --- |
|  | *Dimensions*  *(OD x Length)* | *Tubing* | *Reinforcement* | *Dimensions*  *(OD x Length)* | *Tubing* | *Reinforcement* |  |
| 1 | 2.5 x 50 mm | Natural rubber | Helical coil | 2.5 x 50 mm | Natural rubber | Helical coil | General validations |
| 2 | 2.5 x 200 mm | Natural rubber | Helical coil | 2.5 x 50 mm | Natural rubber | Helical coil | Sensitivity optimization |
| 3 | 2.5 x 35 mm | Natural rubber | Helical coil | 1.5 x 14 mm | Silicone | Helical coil | Soft robotic catheter |
| 4 | 2.5 x 60 mm | Natural rubber | Helical coil | 3 x 35 mm | Natural rubber | Helical coil | Continuum robot |

The wearable controller, on the other hand, has two main parts called hand-glove and forearm-band. The hand glove consists of a 3D-printed TPU part attached to a fabric glove, while the forearm band is 3D printed with TPU solely, where the tuning mechanism is mounted on. Like the desktop device, four master muscles have also been employed and arranged in a circle around the wrist, where one of their ends is fixed on the hand glove and the other end is connected to the tuning mechanism (**Figure 3h** – Main Manuscript).


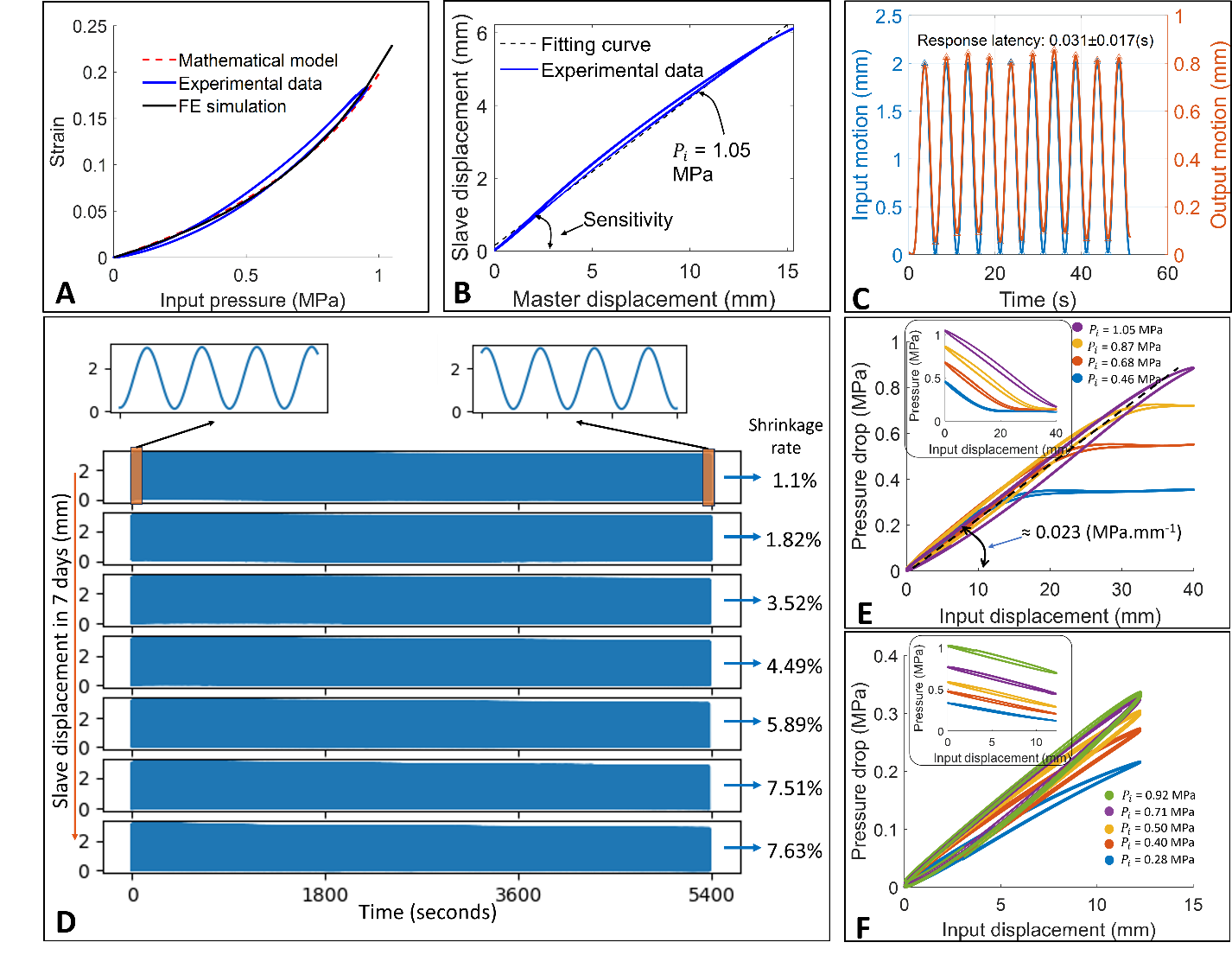


**Figure S3.** (A) Mathematical model, experimental data, and finite element (FE) simulation results of a single fiber-reinforced muscle in terms of strain. (B) Experimental data of master and slave displacements with respect to an initial pressure of 1.05 MPa within a small range of master displacement of 15 mm (30% of the initial length of 50 mm). The method of calculating motion sensitivity. (C) The experimental results of the SFSA characterization for response latency between the input motion of the operator and the output motion of the slave muscle. (D) The results of the SFSA durability test for seven consecutive days and the computed shrinkage rates for each day. (E) Calculated pressure drops versus input displacements from experimental data (in the box) in the case where the end of the slave muscle is free (see the setup in **Figure S5**). (F) Computed pressure drops versus input displacement from experimental data (in the box) in the case where the end of the slave muscle is fixed (see the setup in **Figure S8**).

# **Supplementary Note 2. Modeling and Applications of the SFSA**

## *Modeling of the Single Soft Fiber-Reinforced Muscle (SFRM)*

Here, we consider the wall of the single soft actuator of the SFSA as a composite material with an isotropic hyperelastic matrix and anisotropic embedded fiber. Our approach is based on four key assumptions: 1. Voids in the material and irregularity in the fiber deposition are ignored in this model. 2. This model neglects interaction effects like sliding friction between the fiber and elastomer, and local buckling. 3. The soft actuator's composite material is assumed to be incompressible (with Poisson’s ratio of natural rubber tube approaching 0.5). 4. The deformed soft actuator is assumed to be roughly cylindrical. In this section, we first construct the modeling for a single actuator under pressurization. In the next section, we build the expressions for describing the interaction between the master and slave muscles of the SFSA.

As mentioned above, the helical coil is a single fiber family, which has an angle *α* from the longitudinal axis. When pressurized, the SFRM can potentially change its length (from *L* → *l*), radius (from $R_{i}, R_{o} \to r_{i}, r_{o}$), and rotational angle between one end to the other end (from 0 → *φ*). With the fourth assumption, the transformation of any point in the SFRM wall can be written as a change of cylindrical coordinates from (*R*, *Θ*, *Z)* → (*r*, *θ*, *z)* as shown in **Figure 1b** (Main Manuscript). *R*, *Θ*, *Z*, and *r*, *θ*, *z* are the radial, circumferential, and longitudinal coordinates in the reference and current configurations, respectively. The possible extension, expansion, and twisting deformations are described by:

$r=\sqrt{\frac{R^{2}-R_{i}^{2}}{\frac{l}{L}}+r_{i}^{2}}$ (S1)

$\theta=\Theta+Z\frac{\varphi}{L}$ (S2)

$z=\frac{l}{L}Z=\lambda_{z}Z$ (S3)

From **Equations** **(S1-3)** the deformation gradient **F** can be described as:

$\boldsymbol{F}=\left( r\begin{matrix} \frac{\partial r}{\partial R} & \frac{1}{R}\frac{\partial r}{\partial\Theta} & \frac{\partial r}{\partial Z} \\ \frac{\partial\theta}{\partial R} & \frac{r}{R}\frac{\partial\theta}{\partial\Theta} & r\frac{\partial\theta}{\partial Z} \\ \frac{\partial z}{\partial R} & \frac{1}{R}\frac{\partial z}{\partial\Theta} & \frac{\partial z}{\partial Z} \end{matrix} \right)=\left( \begin{matrix} \frac{R}{r\lambda_{z}} & 0 & 0 \\ 0 & \frac{r}{R} & \frac{r\varphi}{L} \\ 0 & 0 & \lambda_{z} \end{matrix} \right)$ (S4)

The deformation gradient ***F*** is then used to calculate the left and right Cauchy-Green deformation tensor $\boldsymbol{B}\boldsymbol{=}\boldsymbol{F}\boldsymbol{F}^{T}$ and the first tensor invariant as follows:

$I_{1}=tr\left( \boldsymbol{B} \right)\boldsymbol{=}\lambda_{z}^{2}+\frac{r^{2}}{R^{2}}+\frac{R^{2}}{\lambda_{z}^{2}r^{2}}+\frac{r^{2}\varphi^{2}}{L^{2}}$ (S5)

We defined a unit vector $\boldsymbol{S}={[0 sin(\alpha) cos (\alpha)]}^{T}$ tangent to the helical coil in the SFRM’s initial configuration, where $\alpha$ is the angle between the fiber and the longitudinal axis, as shown in **Figure 1b** (Main Manuscript). When the SFRM deforms, the fiber changes, which can be described by the deformed fiber direction **s**:

$\boldsymbol{s}=\boldsymbol{FS}=\left[ \begin{matrix} 0 \\ \frac{r}{R}\sin\left( \alpha\right)+\frac{r\varphi}{L}cos(\alpha) \\ \lambda_{z}cos(\alpha) \end{matrix} \right]$ (S6)

From^[4]^ the fourth tensor invariant can be obtained as:

$I_{4}=\mathbf{s}.\mathbf{s}=\lambda_{z}^{2}\cos^{2} \left( \alpha\right)+\frac{r^{2}\varphi^{2}\cos^{2} \left( \alpha\right)}{L^{2}}+2\frac{r^{2}\varphi\cos\left( \alpha\right)\sin\left( \alpha\right)}{LR}+\frac{r^{2}\sin^{2} (\alpha)}{R^{2}}$ (S7)

In terms of strain energy for the SFRM, we can find the total strain energy through the superposition of elastomer and fiber energy as follows:

$\Psi=\Psi_{tube}+\Psi_{fiber}$ (S8)

For the inner tube, we choose a simple incompressible Neo-Hookean model:

$\Psi_{tube}=\frac{\mu_{1}}{2}(I_{1}-3)$ (S9)

where $\mu_{1}$ denotes the initial material property of the inner tube.

Since the helical coil is a linear spring, we applied the same method in^[5]^ to derive the expression for $\Psi_{fiber}$. In which, we consider a small segment of the helical fiber of length *dl* which has a change in length *dx* undergoes an axial load $F_{f}$. The axial load of this linear elastomer fiber can be deprived as $F_{f}={A\mu}_{2}\varepsilon$, where *A* and *μ*_2_ are the cross-sectional area and the material parameter of the fiber, respectively; and $\varepsilon=\frac{dx}{dl}$ is the axial strain. So that the strain energy for this segment is equal to the area underneath the force-displacement curve, which can be described as:

$dW_{fiber}=\frac{1}{2}F_{f}dx=\frac{1}{2}{A\mu}_{2}\varepsilon^{2}dl$ (S10)

Integrating **Equation** **(S10)**, we have the energy of the entire helical coil of length *L_f_*:

$W_{fiber}=\int_{0}^{L_{f}} \frac{1}{2}{A\mu}_{2}\varepsilon^{2}dl=\frac{1}{2}{A\mu}_{2}\varepsilon^{2}L_{f}$ (S11)

As the physical meaning of the fourth invariant *I_4_* is the square of the stretch in the referred direction ***s***^[6]^, we have $I_{4}=\lambda_{f}^{2}=\left( \frac{dx+dl}{dl} \right)^{2}$ yielding $\varepsilon=\sqrt{I_{4}}-1$, where $\lambda_{f}$ is the stretch along the fiber direction. Substituting this equation into **Equation** **(S11)** and dividing by the volume of the helical coil yields the strain energy density:

$\Psi_{fiber}\boldsymbol{=}\frac{\mu_{2}}{2}\left( \sqrt{I_{4}}-1 \right)^{2}$ (S12)

Substituting **Equations** **(S9)** and **(S12)** into **Equation** **(S8)** yields:

$\Psi=\frac{\mu_{1}}{2}(I_{1}-3)+\frac{\mu_{2}}{2}\left( \sqrt{I_{4}}-1 \right)^{2}$ (S13)

This total strain energy is then used to calculate the Cauchy stress as follows:

$\boldsymbol{\sigma}=\left[ \begin{matrix} \sigma_{rr} & \sigma_{r\theta} & \sigma_{rz} \\ \sigma_{\theta r} & \sigma_{\theta\theta} & \sigma_{\theta z} \\ \sigma_{zr} & \sigma_{z\theta} & \sigma_{zz} \end{matrix} \right]=\frac{\partial\Psi}{\partial F}\boldsymbol{F}^{T}\boldsymbol{-}p\boldsymbol{I}$ (S14)

Where *p* is a hydrostatic pressure ^[7]^ and ***I*** is the identity matrix. Further expanding **Equation** **(S14)**, we have:

$\boldsymbol{\sigma=}\frac{\partial\Psi_{tube}+\partial\Psi_{fiber}}{\partial\boldsymbol{F}}\boldsymbol{F}^{T}\boldsymbol{-}p\boldsymbol{I}$

$\boldsymbol{=}\frac{\partial\Psi_{tube}\partial I_{1}}{\partial I_{1}\partial\boldsymbol{F}}\boldsymbol{F}^{T}+\frac{\partial\Psi_{tube}\partial I_{4}}{\partial I_{4}\partial\boldsymbol{F}}\boldsymbol{F}^{T}-p\boldsymbol{I}$

$\boldsymbol{=}\mu_{1}\boldsymbol{B}+\mu_{2}\frac{\sqrt{I_{4}}-1}{\sqrt{I_{4}}}\boldsymbol{s\bigotimes s}\mathbf{-}p\boldsymbol{I}$ (S15)

To determine the current muscle configuration, we applied the Cauchy equilibrium equation:^[4-5]^

$\nabla. \boldsymbol{\sigma}\boldsymbol{=}0$ (S16)

In cylindrical coordinates, **Equation** **(S16)** can be expressed as:

$\frac{\partial\sigma_{rr}}{\partial r}+\frac{1}{r}\frac{\partial\sigma_{r\theta}}{\partial\theta}+\frac{\partial\sigma_{rz}}{\partial z}+\frac{1}{r}\left( \sigma_{rr}-\sigma_{\theta\theta} \right)=0$

$\frac{\partial\sigma_{r\theta}}{\partial r}+\frac{1}{r}\frac{\partial\sigma_{\theta\theta}}{\partial\theta}+\frac{\partial\sigma_{\theta z}}{\partial z}+\frac{2}{r}\sigma_{r\theta}=0$ (S17)

$\frac{\partial\sigma_{rz}}{\partial r}+\frac{1}{r}\frac{\partial\sigma_{\theta z}}{\partial\theta}+\frac{\partial\sigma_{zz}}{\partial z}+\frac{1}{r}\sigma_{rz}=0$

Due to axial symmetry and uniform stretch of the SFRM, there is none of the stresses vary in the θ or z directions ($\sigma_{r\theta}= \sigma_{rz}=0$). Therefore, we consider the first hydrostatic equilibrium equation, which can be re-arranged as:

$\frac{\partial\sigma_{rr}}{\partial r}=\frac{1}{r}(\sigma_{\theta\theta}-\sigma_{rr})$ (S18)

Integrating **Equation (S18)** in *r* with the boundary conditions $\sigma_{rr}\left( r_{i} \right)=-P$ and $\sigma_{rr}(r_{o})=0$; and applying a variable change from *r*-to-*R* yields:

$P= \int_{r_{i}}^{r_{o}} \frac{1}{r}\left( \sigma_{\theta\theta}-\sigma_{rr} \right)dr$

$= \int_{R_{i}}^{R_{o}} \frac{1}{r}\left( \sigma_{\theta\theta}-\sigma_{rr} \right)\frac{dr}{dR}dR$

$= \int_{R_{i}}^{R_{o}} \frac{1}{r}\left( \sigma_{\theta\theta}-\sigma_{rr} \right)\frac{R}{r\lambda_{z}}dR$

$= \int_{R_{i}}^{R_{o}} \frac{R}{{(R}^{2}-R_{i}^{2}+\lambda_{z}r_{i}^{2})}\left( \sigma_{\theta\theta}-\sigma_{rr} \right)dR$ (S19)

where *P* is the inner pressure applied inside the tube and $\sigma_{\theta\theta}-\sigma_{rr}$ can be calculated from the Cauchy stress in **Equation (S15)**. Since there are no external axial forces or external axial moments applied to the single SFHM, the axial load *N* and the axial moment *M* can be expressed as follows:

$N= 2\pi\int_{r_{i}}^{r_{o}} \sigma_{zz}rdr=P\pi r_{i}^{2}$ (S20)

$M= 2\pi\int_{r_{i}}^{r_{o}} \sigma_{\theta z}r^{2}dr=0$ (S21)

**Equation (S20)** can also be re-arranged as follows:

$0=2\pi\int_{r_{i}}^{r_{o}} \sigma_{zz}rdr-P\pi r_{i}^{2}$

$=2\pi\int_{r_{i}}^{r_{o}} (\sigma_{zz}-\sigma_{rr}+\sigma_{rr})rdr$

$=2\pi\int_{r_{i}}^{r_{o}} \left( \sigma_{zz}-\sigma_{rr} \right)rdr+\left. \pi\sigma_{rr}r^{2} \right|_{r_{i}}^{r_{o}}-\pi\int_{r_{i}}^{r_{o}} \frac{r^{2}d\sigma_{rr}}{dr}-P\pi r_{i}^{2}$

$=2\pi\int_{r_{i}}^{r_{o}} \left( \sigma_{zz}-\sigma_{rr} \right)rdr-\pi\int_{r_{i}}^{r_{o}} \frac{r^{2}}{r}(\sigma_{\theta\theta}-\sigma_{rr}) dr$

$=\pi\int_{r_{i}}^{r_{o}} \left( 2\sigma_{zz}-\sigma_{rr}-\sigma_{\theta\theta} \right)rdr$

$=\pi\int_{R_{i}}^{R_{o}} \left( 2\sigma_{zz}-\sigma_{rr}-\sigma_{\theta\theta} \right)r\frac{dr}{dR}dR$

$=\pi\int_{R_{i}}^{R_{o}} \left( 2\sigma_{zz}-\sigma_{rr}-\sigma_{\theta\theta} \right)\frac{R}{\lambda_{z}}dR$ (S22)

While **Equation (S21)** can be converted as follows:

$0=2\pi\int_{r_{i}}^{r_{o}} \sigma_{\theta z}r^{2}dr$

$=2\pi\int_{R_{i}}^{R_{o}} \frac{\sigma_{\theta z}r^{2}dr}{dR}dR$

$=2\pi\int_{R_{i}}^{R_{o}} \frac{\sigma_{\theta z}rR}{\lambda_{z}}dR$ (S23)

By solving **Equations (S19)**, **(S22)**, and **(S23)**, we can find three unknown parameters including $r_{i}$, *l*, and *φ*, therefore, we can predict the elongation of a single SFRM under an input pressure *P*, which is the initial information for solving the model of the soft syringe in the next section. We experimented to validate the model of the single muscle, as the result shown in **Figure S3A**, the proposed model is neatly fitted with the experimental data. **Table S2** describes the input parameters for solving the model.

**Supplymentary Table S2. Input parameters for the SFSA model**

| **Input parameters** | **Symbol** | **Value** | **Unit** |
| --- | --- | --- | --- |
| Initial length | *L* | 0.05 | m |
| Initial inner radius | *R_i_* | 0.00035 | m |
| Initial outer radius | *R_o_* | 0.0023 | m |
| Initial twisting angle | *Θ* | 0 | rad |
| Initial fiber-direction angle | *α* | 1.44 | rad |
| Initial shear modulus of  the inner tube | *μ_1_* | 427560 | Pa |
| Material parameter of the fiber | *μ_2_* | 8500000 | Pa |
| Pressure | *P* | from 0 to 1000000 | Pa |

## *Modeling of SFSA*

Here, we consider the simple frame of an SFSA using a pair of twin SFRMs, with an assumption that the two muscles have the same sizes and perform exactly alike. Under pressure *P*, the two muscles elongate to a length $l_{P}$, expand to the inner radius $r_{iP}$, and twist to $\varphi_{P}$. The pressure is held in this state. One muscle called the master muscle, is dragged to the length $l_{a}$, which theoretically increases its volume consequently reducing the inner pressure of the entire SFSA. The other muscle, called the slave muscle, is, therefore, shortened to the length $l_{b}$*.* It is worth noting that the master muscle is unable to be twisted as we must hold one end to drag it, therefore it keeps its twisting angle $\varphi_{P}$ during this action. Meanwhile, the slave muscle is free to elongate, expand, and twist.

To find the relationship between the input displacement and the output displacement, we applied the boundary conditions to establish four equations with four unknown parameters. Firstly, the pressure inside the master and slave muscles is equal at the equilibrium state. Extending **Equation (S19)** for two muscles yields:

$\int_{R_{i}}^{R_{o}} \frac{R_{a}}{{(R}_{a}^{2} -R_{i}^{2}+\frac{l_{a}}{L}r_{ia}^{2})}\left( \sigma_{\theta\theta a}-\sigma_{rra} \right)dR_{a}= \int_{R_{i}}^{R_{o}} \frac{R_{b}}{{(R}_{b}^{2}-R_{i}^{2}+\frac{l_{b}}{L}r_{ib}^{2})}\left( \sigma_{\theta\theta b}-\sigma_{rrb} \right)dR_{b}$ (S24)

where $r_{ia}$ and $r_{ib}$ denote the inner radii, and $\sigma_{\theta\theta a}, \sigma_{rra}, \sigma_{\theta\theta a}$, and $\sigma_{rrb}$ are the stresses of the two muscles at the equilibrium state; $R_{a}$ and $R_{b}$ are the radii in the reference configurations of the two muscles.

Second, the volume inside the master muscle increased and the liquid volume technically moves from the slave muscle to the master muscle, hence the change of volume in these two muscles are equal.

$l_{a}r_{ia}^{2}-l_{P}r_{iP}^{2}=l_{P}r_{iP}^{2}-l_{b}r_{ib}^{2}$ (S25)

Since the slave muscle are free, the last two equations are similar to **Equations (S22)** and **(S23)**, but applying for the slave muscle:

$\pi\int_{R_{i}}^{R_{o}} \left( 2\sigma_{zzb}-\sigma_{rrb}-\sigma_{\theta\theta b} \right)\frac{{LR}_{b}}{l_{b}}dR_{b}=0$ (S26)

$2\pi\int_{R_{i}}^{R_{o}} \frac{{L\sigma}_{\theta zb}r_{b}R_{b}}{l_{b}}dR=0$ (S27)

where $\sigma_{\theta zb}$ and $\sigma_{zzb}$ denote the stresses, and $r_{b}$ is the radius in the current configuration of the slave muscle.

Solving **Equations (S24)**, **(S25)**, **(S26)**, and **(S27)** we can obtain four unknown parameters including $l_{b}, r_{ib}, \varphi_{b}$, and $r_{ia}$, in other words, we can predict the output behaviors of the slave muscle from the input conditions of the master muscle. There are six parameters linked together $l_{a}, l_{b}, r_{ia}, r_{ib}, \varphi_{a}$, and $\varphi_{b}$, thus we need six equations to solve. Technically, we can have two equations from the inner pressure balance of the SFSA and the conservation of liquid volume inside the system. Therefore, for each problem, if we can have four other equations based on the input conditions, we will be able to mathematically predict the behaviors of the slave muscle.

## *Finite Element (FE) Simulations of SFSA*

A 3D simulation was set up to model two short segments (5 mm) of a pair of slave and master muscles using COMSOL Multiphysics 6.0.0.405. The purpose of this simulation is to provide another designing means, where it can simply highlight the expected trends and relationships between the master and slave muscles when changing the modeled parameters. Additionally, we used a volumetric constraint method (VCM) instead of Fluid-Structure Interaction (FSI) to reduce simulation time and complexity, as well as the need for computational resources. Therefore, the material parameters were chosen differently from the mathematical model and were not characterized yet. However, they were chosen to ensure that the relationship between elongation and input pressure during the initial phase (Phase 1) of the SFSA activation closely matched the mathematical and experimental data (**Figure S3A**). Specifically, we used $\mu_{1}=$2.85 Mpa and $\mu_{2}=$1 GPA as the material parameters for the rubber tube and fiber-reinforcement layer, respectively. **Figure S4** shows the setup of the FE simulation for the SFSA. At the initial state, the pressure is applied to the inner surface of the two small segment muscles, while the prescribed displacement in the X and Y directions is applied to prevent the bending of the muscle. Two bottom parts were assigned as stainless-steel material and fixed, while the active segments were free to elongate, twist, and radially expand. After Phase 1, the inner volume is measured, and a pulling load is applied to the top surface of the master muscle (on the left). In the second phase, while the displacement was applied to the end of the master muscle, the applied pressure was updated step-by-step to ensure the fluid volume stayed constant throughout the simulation period, analogous to a proportional controller.


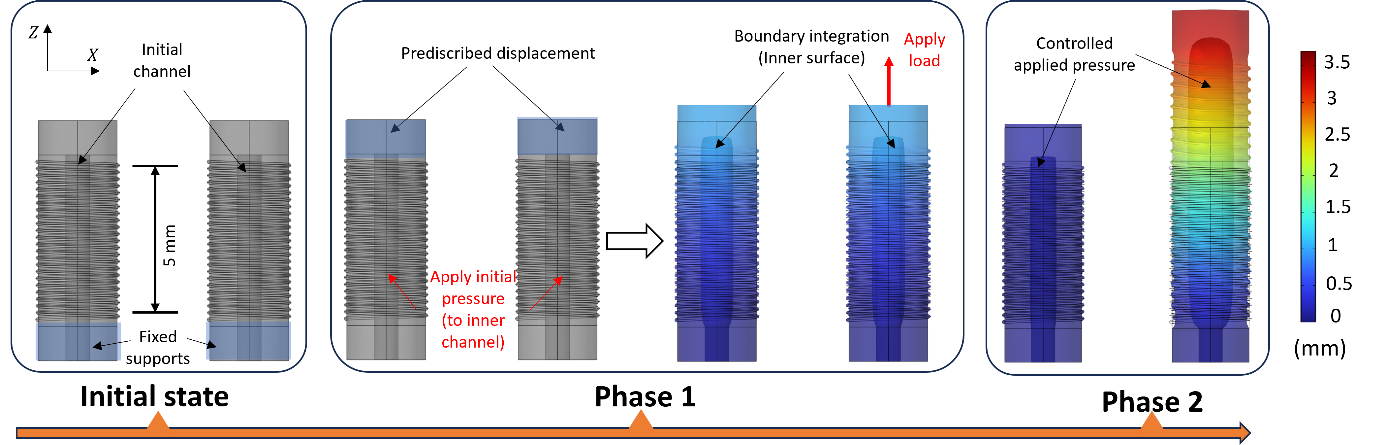


**Figure S4.** The setup of FE simulation for the small segments of the SFSA.

## *Analytical Modeling of the Soft Robotic Catheter*

**Figure S2B** shows the perpendicular projections of the desktop device with simplifications on XY and XZ planes. While the Z-axis is defined as the central axis of the four master muscles, the Y-axis is the vertical axis, and the X-axis is perpendicular to the YZ plane. As four muscles have been arranged 90^o^ apart, the length of the $i^{th}$ master muscle ($i=1, 2, 3, 4$) can be described as:

$l_{mi}=R_{dt}\sin\left[ \gamma_{1}+\frac{\left( i-1 \right)\pi}{2} \right]cos\gamma_{2}+l_{m}$ (S28)

where $\gamma_{1}$ and $\gamma_{2}$ are respectively the azimuth angle of the plate (the angle between the Y-axis and the first muscle) and the tilted angle of the plate (the angle between the plate and X-axis), respectively. $R_{td}$ is the radius of the muscles-arranged circle and $l_{m}$ denotes the initial length of each muscle. Substitute $i=1, 2, 3, 4$, yields:

$\left[ \begin{aligned} l_{m1} \\ l_{m2} \\ l_{m3} \\ l_{m4} \end{aligned} \right]=\left[ \begin{aligned} {R_{td}sin\gamma_{1}cos\gamma_{2}+l}_{m} \\ {R_{td}cos\gamma_{1}cos\gamma_{2}+l}_{m} \\ {{-R}_{td}sin\gamma_{1}cos\gamma_{2}+l}_{m} \\ {{-R}_{td}cos\gamma_{1}cos\gamma_{2}+l}_{m} \end{aligned} \right]$ (S29)

Here, we simplified the SRA by assuming that the slave muscles of the SRA have negligible effects on each other when their lengths are changed. Applied the model of the SFSA depicted in the above section, we obtain the corresponding length of the slave muscles, which can be described as:

$\left[ \begin{aligned} l_{s1} \\ l_{s2} \\ l_{s3} \\ l_{s4} \end{aligned} \right]=F_{SFSA}\left( P_{initial},\left[ \begin{aligned} l_{m1} \\ l_{m2} \\ l_{m3} \\ l_{m4} \end{aligned} \right] \right)$ (S30)

where $F_{SFSA}$ is the function of the SFSA model with the inputs being the initial pressure and the instantaneous lengths of the master muscles. The initial pressure here also plays the role of a scaling factor or motion sensitivity factor. Technically, once a difference in length between slave muscle appears, the SRA starts bending, which can be identified by three parameters including the bending radius $R_{arm}$ or curvature, the angle of the bending plane with respect to the X-axis $\delta_{1}$, and the bending angle of the arc of the SRA $\delta_{2}$:^[8]^

$R_{arm}= \frac{d(l_{s1}+l_{s2}+l_{s3}+l_{s4})(l_{s4}-l_{s2})}{(l_{s1}-3l_{s2}+l_{s3}+l_{s4})\sqrt{\left( l_{s4}-l_{s2} \right)^{2}+\left( l_{s3}-l_{s1} \right)^{2}}}$ (S31)

$\delta_{1}={tan}^{-1}\left( \frac{l_{s4}-l_{s2}}{l_{s3}-l_{s1}} \right)$ (S32)

$\delta_{2}=\frac{(l_{s1}-3l_{s2}+l_{s3}+l_{s4})\sqrt{\left( l_{s4}-l_{s2} \right)^{2}+\left( l_{s3}-l_{s1} \right)^{2}}}{4d(l_{s4}-l_{s2})}$ (S33)

where *d* is denoted as the distance from the central point of the arm to the central point of a muscle on the end surface. From here, the motions of the catheter can be predicted with respect to the change in the desktop device. If we consider the wrist arm as a ball joint, the model for the wearable controller would be the same as that of the desktop device. **Equations (S31-33)** represent the standard kinematic model of the constant curvature continuum, however, singularities and discontinuities are its limitations. In this application, these limitations happened when the subtractions ${\Delta_{y}=l}_{s4}-l_{s2}$and $\Delta_{x}=l_{s3}-l_{s1}\to0$, making the visual feedback of the catheter motion discontinue and not fit with the real motion of the catheter. Therefore, we adopted an optimization model developed by Cosimo et al.^[9]^ to cover this issue, which can simply be described as follows:

$\delta_{1}=sign\left( \Delta_{y} \right)\cos^{-1} \left( \frac{\Delta_{x}}{\Delta} \right)$ (S34)

$\delta_{2}=\frac{\Delta}{R_{dt}}$ (S35)

$R_{arm}=\frac{l_{s}}{\delta_{2}}$ (S36)

where $\Delta=\sqrt{\Delta_{x}^{2}+\Delta_{y}^{2}}$ and $l_{s}=\frac{l_{s1}+l_{s2}+l_{s3}+l_{s4}}{4}$. By using this model with input parameters described in **Table S3**, the workspace of the end-effector (**Figure S2C**) and the real-time visual feedback of the SRA’s motions were obtained and shown in **Movie S2**.

**Supplementary Table S3**. **Input parameters for the model of the soft robotic catheter**

| **Input parameters** | | **Symbol** | **Value** | **Unit** |
| --- | --- | --- | --- | --- |
| Radius of muscle-arranged circle | | *R_td_* | 0.00165 | m |
| Azimuth angle | | *γ_1_­* | 0 | rad |
| Tilt angle | | *γ_2_* | (0- 0.1745) | rad |
| The central-to-central distance of the arm and the muscle. | | *d* | 0.0011 | m |
| Initial pressure | | *P_i_* | 900000 | Pa |
| The master muscle | Initial length | *L_m_* | 0.035 | m |
|  | Initial inner radius | *r_im_* | 0.00035 | m |
|  | Initial outer radius | *R_om_* | 0.0023 | m |
|  | Initial twisting angle | *Θ* | 0 | rad |
|  | Initial fiber-direction angle | *α* | 1.44 | rad |
|  | Initial shear modulus of  the inner tube | *μ_1m_* | 427560 | Pa |
|  | Material parameter of the fiber | *μ_2m_* | 8500000 | Pa |
| The slave muscle | Initial length | *L_s_* | 0.0125 | m |
|  | Initial inner radius | *r_is_* | 0.0002 | m |
|  | Initial outer radius | *R_os_* | 0.0007 | m |
|  | Initial twisting angle | *Θ_s_* | 0 | rad |
|  | Initial fiber-direction angle | *α_s_* | 1.44 | rad |
|  | Initial shear modulus of  the inner tube | *μ_1s_* | 235680 | Pa |
|  | Material modulus of the fiber | *μ_2s_* | 8500000 | Pa |

# **Supplementary Note 3. Experimental setups for characterizations of the SFSA**


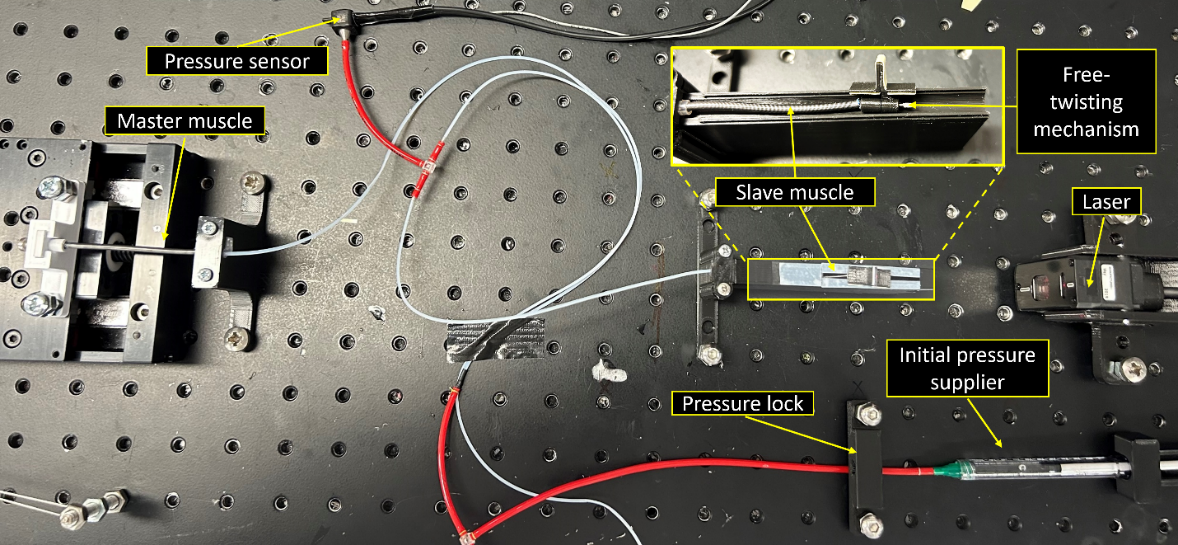


**Figure S5.** The experimental set-up for general characterizations of the SFSA fundamental frame including motion validation, durability, and response latency tests.


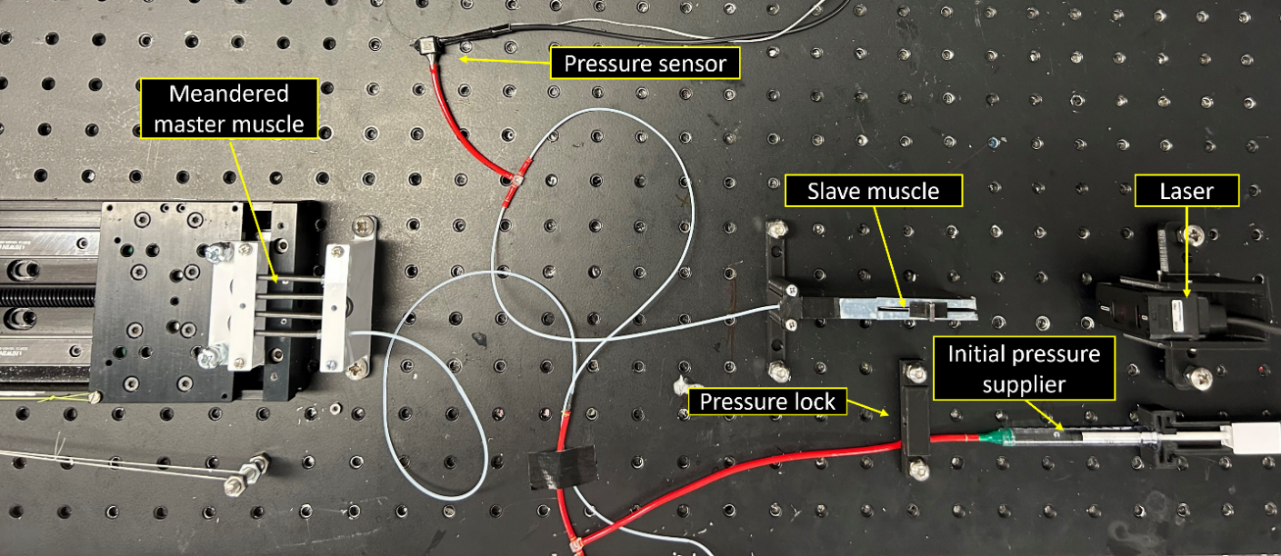


**Figure S6.** The experimental set-up for the test of meandering to increase the SFSA’s motion sensitivity.


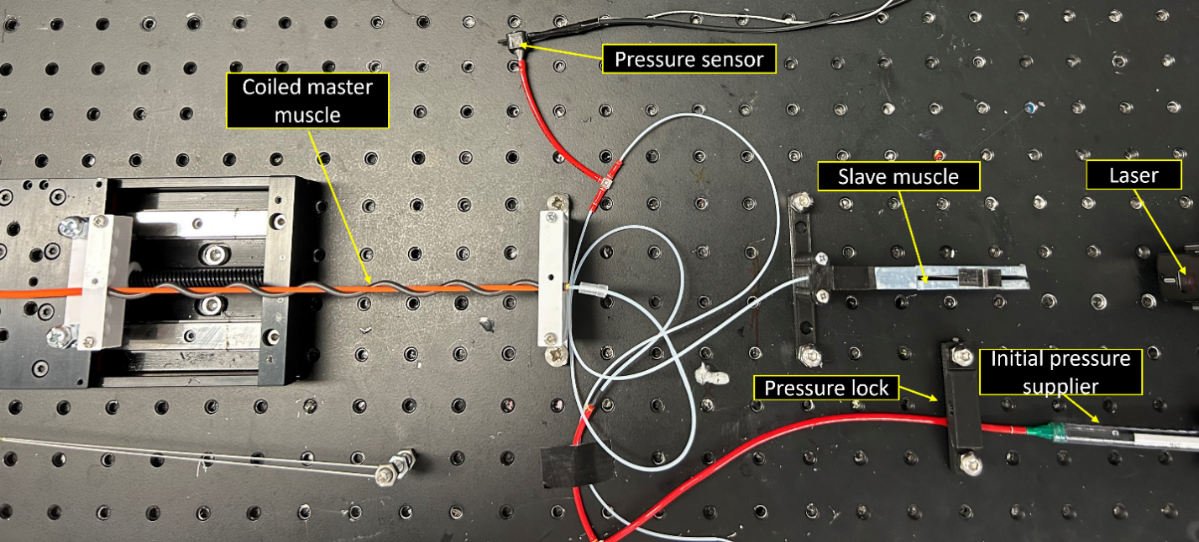


**Figure S7.** The experimental set-up for the test of coiling to decrease the SFSA’s motion sensitivity.


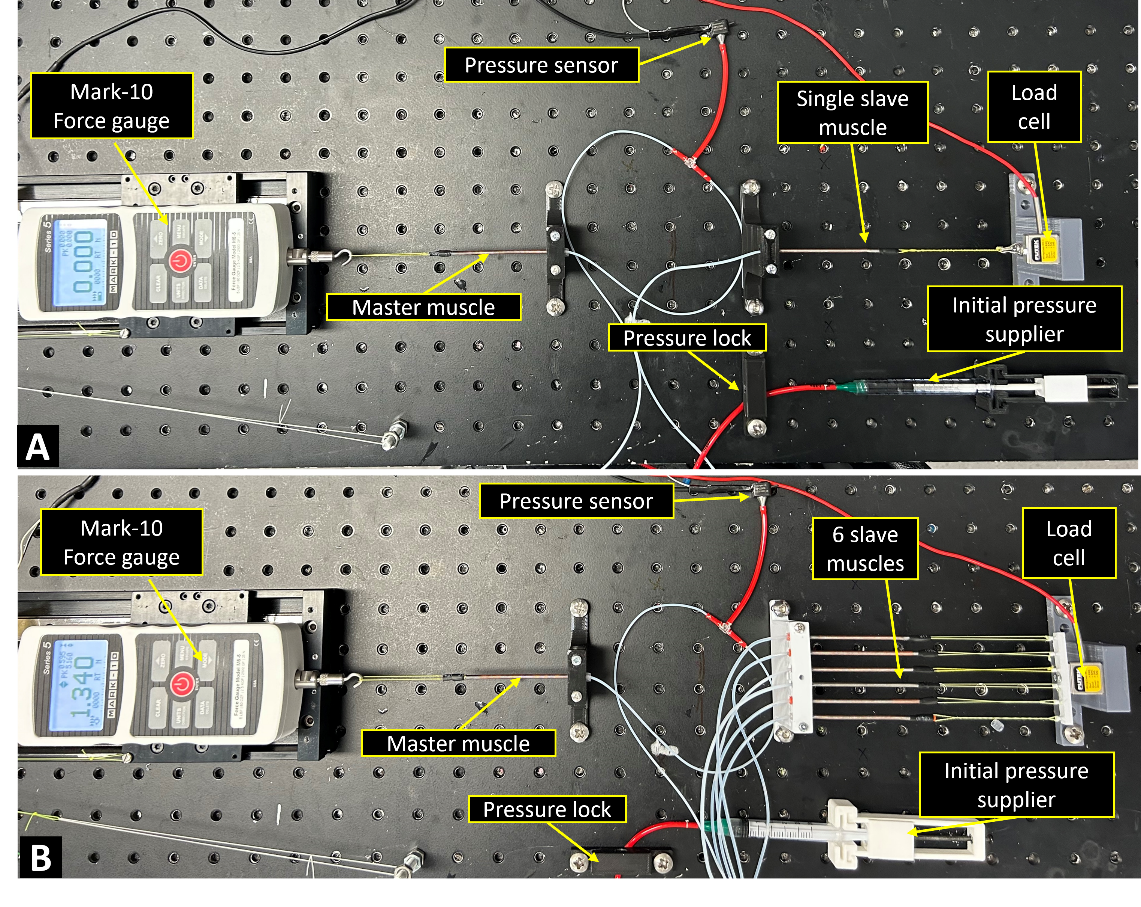


**Figure S8.** The experimental set-up for force characterizations of the SFSA with (A) a single slave muscle and (B) more slave muscles added.

# **Supplementary Note 4. Characterizations and demonstrations of the soft robotic catheter**

## *Motion Characterizations*


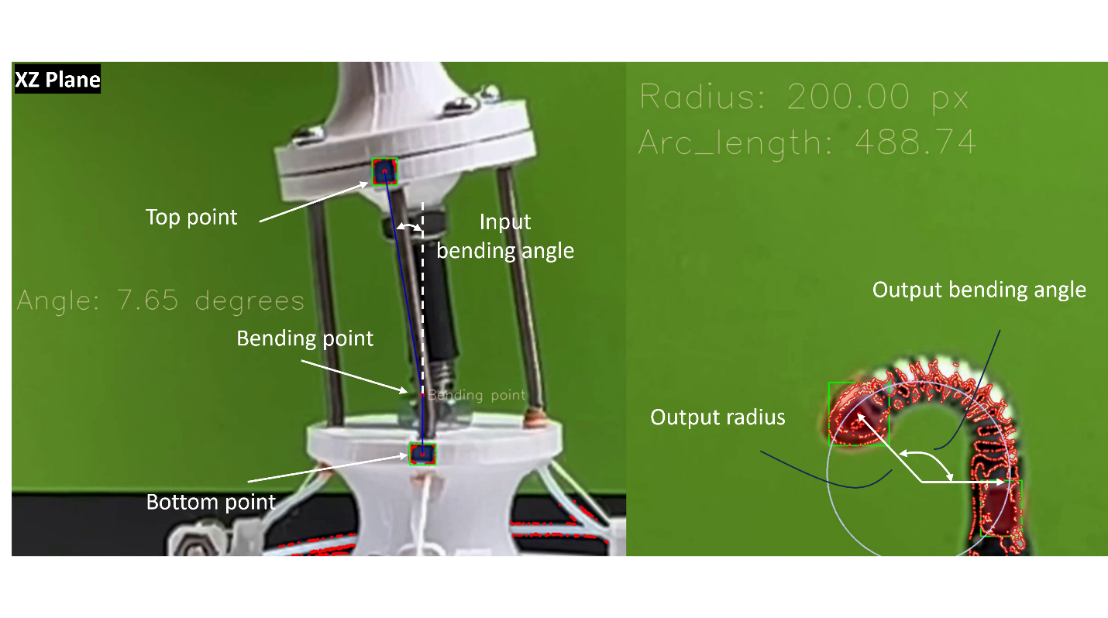


**Figure S9.** Image processing with OpenCV is used to characterize the soft robotic catheter system with the desktop controller.

Characterizations of Passive and Active Sensing Sensitivities

Characterizations for the *passive sensing* sensitivity of the soft catheter within four initial shapes including a straight shape (with equal initial pressure of 0.6 MPa in all four SFSAs) and bending shapes with 15, 30, and 45 degrees, have been performed in this study. The soft catheter was moved down along the Z-axis to touch a load cell (ATI Nano 17, ATI Industrial Automation, Inc., USA), which was used to record the contact force (**Figure S10 –** right panel). At the same time, the pressure of SFSAs was measured by external pressure sensors (**Figure S1F**). The results shown in **Figure S10** (with raw data on the left panel and computed pressure change on the right panel) indicate that the sensitivities of four SFSAs are about the same approximately 0.025 MPa.N^-1^ in the case of a straight shape. However, when the catheter has a bending shape (i.e., SFSAs’ initial pressure is distinct), differences can be observed between the sensitivities of the SFSAs. Specifically, the SFSA with the lowest initial pressure (i.e., the catheter bent towards it), in which SFSA 3 in this test had the highest sensitivity. The disparency is more obvious when the bending angle increases; at 45 degrees, the sensitivity of SFSA 3 is about 0.14 MPa.N^-1^, while the three other SFSAs have sensitivities lower than 0.055 MPa.N^-1^. This finding clearly explains the pressure change of SFSA 2 is higher than that of other SFSAs as shown in **Figure 4G-H** (of the main manuscript). This characterization focuses on the normal contacting force. Notably, this soft catheter is also capable of monitoring the force direction; for example, **Figure S11** shows the test of another direction, exhibiting different pressure changes compared to those shown in **Figure 4G**. This ability of the SFSA-based devices will be explored in future work.


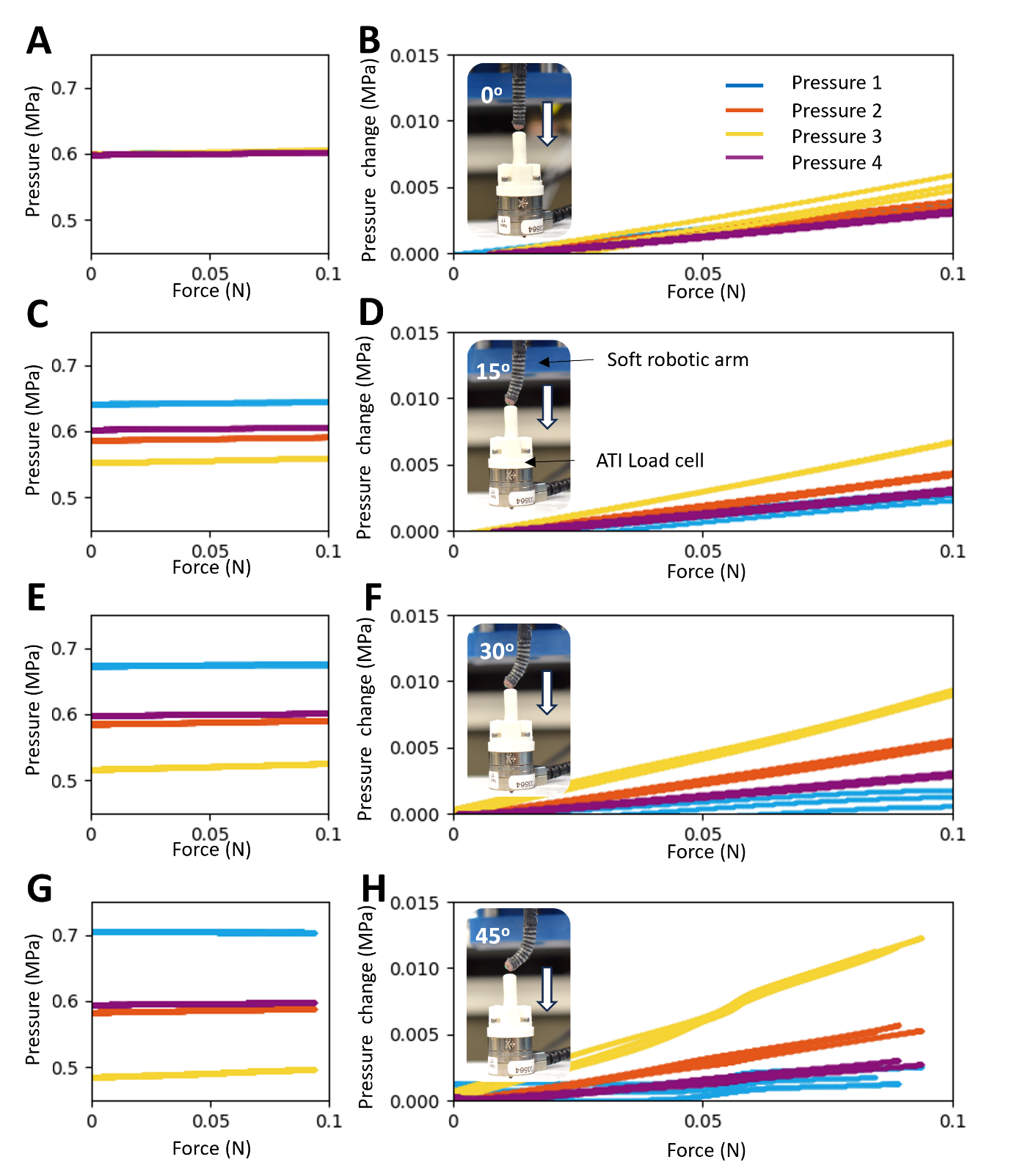


**Figure S10.** Characterizations of the soft robotic catheter in terms of passive touch sensing. (left) Measured pressure of four SFSA channels; (right) experimental setup, and computed pressure changes: (A-B) Straight touch. (C-D) 15-degree-bending touch. (E-F) 30-degree-bending touch. (G-H) 45-degree-bending touch.


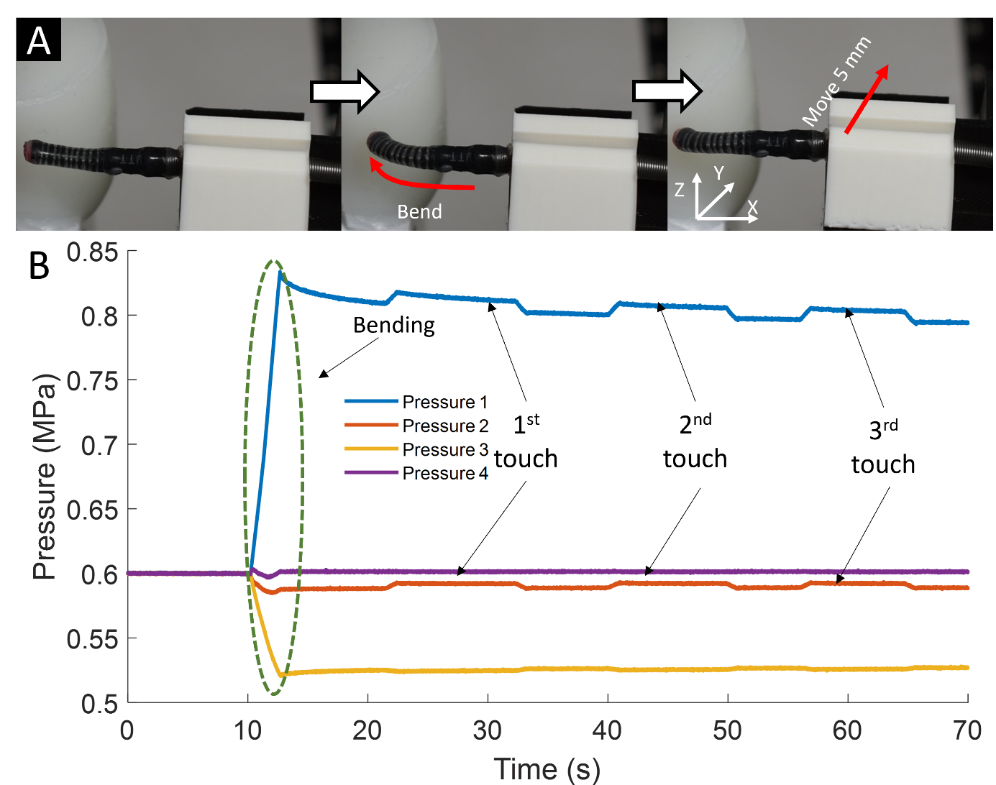


**Figure S11.** Demonstrations of the SFSA’s passive sensing capabilities within the soft robotic catheter: Motions of the soft robotic catheter interacting with the tissue phantom, (A) where the catheter is bent by activating SFSAs 1 and 3 and moved 5 mm along the Y-axis to touch with the tissue phantom in three cycles. (B) Experimental data of the inner pressure during the process (**Movie S8**).

We also characterized the active touch sensing ability of the soft robotic catheter by comparing the pressure when it was bent in free space and when activated in contact with the tip of a sensitive load cell (LVS-5GA – Kyowa Electronic Instruments Co., Ltd., Japan), using the same input motion of the master controller (**Figure S12A**). The results of pressure change are presented in a 3D plot against the input motion and measured force (**Figure S12A**), as well as in 2D plots (**Figure S12B-C**) separately. The average pressure change at the ending points where the input motion reaches max has been computed for each SFSA and then divided by the max force measured by the load cell. As a result, SFSAs 3 and 4 exhibited equal sensitivities of about 0.125 MPa.N^-1^, while SFSAs 1 and 2 showed opposite sensitivities of about -0.125 MPa.N^-1^. Combined with the demonstrations shown in **Figure 4I-J**, we can conclude that the pressure within the SFSAs exhibits distinct changes when the catheter encounters an external object compared to free-space bending under identical input conditions. This pressure variation consistently follows the current trend of either increasing or decreasing. This information is critical for providing real-time feedback to users, indicating contact with an external object, and offering quantitative estimates of the contact force.


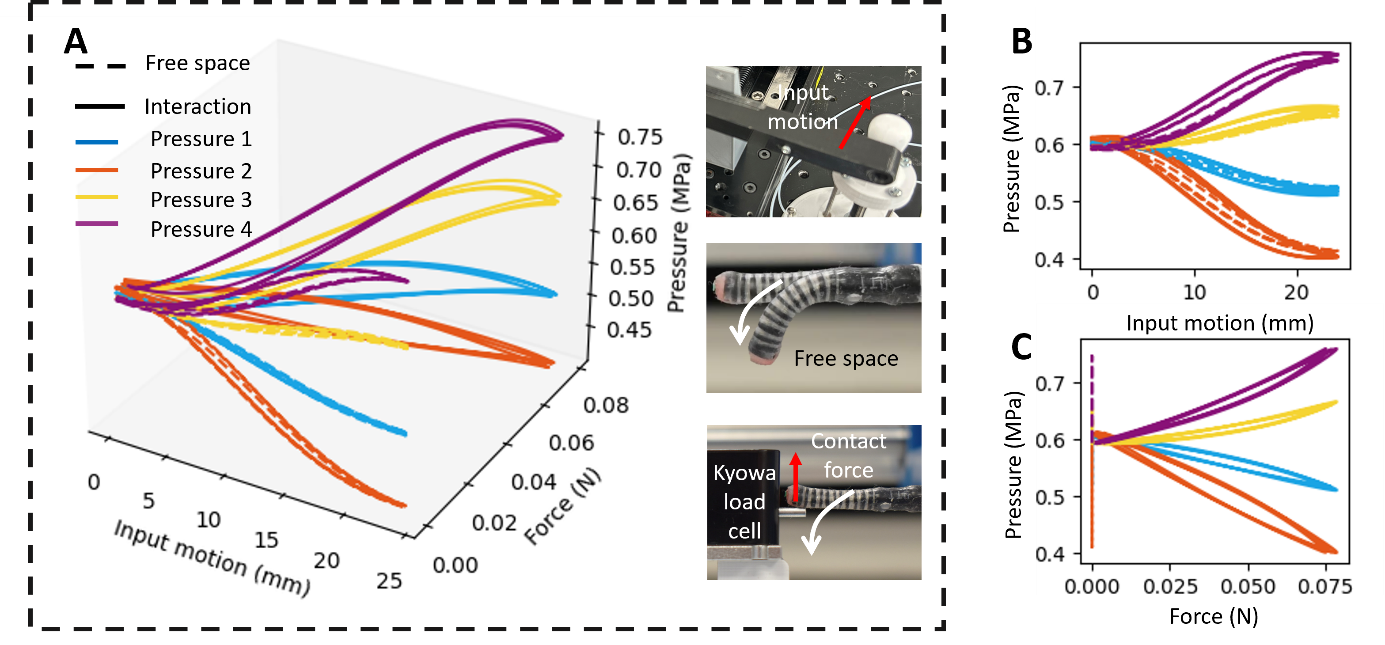


**Figure S12.** Characterizations of the soft robotic catheter in terms of active touch sensing. (A) A 3D plot of the change of the pressure and recorded force against the input motion from the master controller, in free space and contact with the Kyowa load cell tip and the experimental setup. (B) A 2D plot of the pressure change against the input motion. (C) A 2D plot of the pressure change versus the recorded force.

Surface Scanning/Mapping


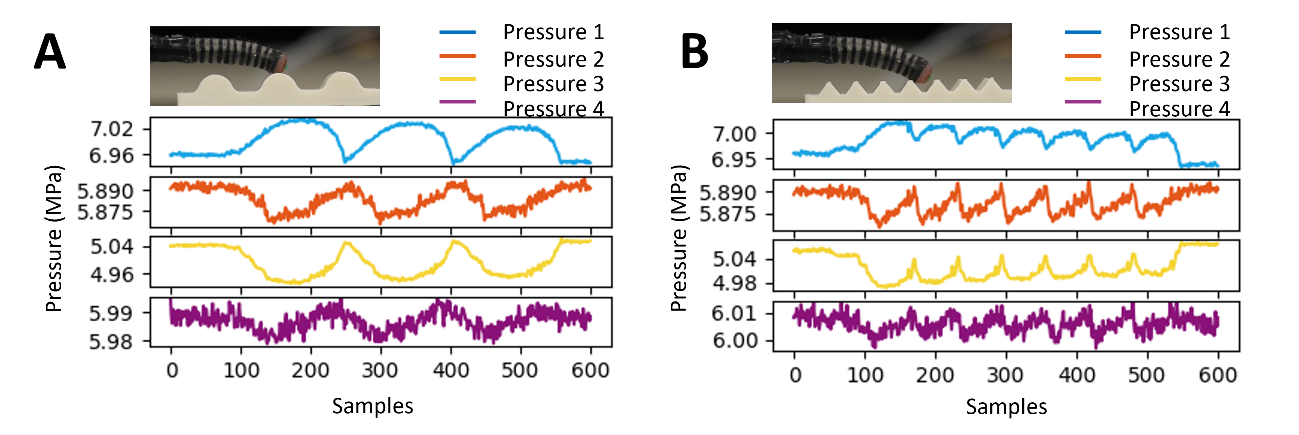


**Figure S13.** Demonstrations of surface scanning and texture detection. Experimental results of scanning the profile of (A) a round-circle surface and (B) a spiked triangle surface.

*Texture detection*


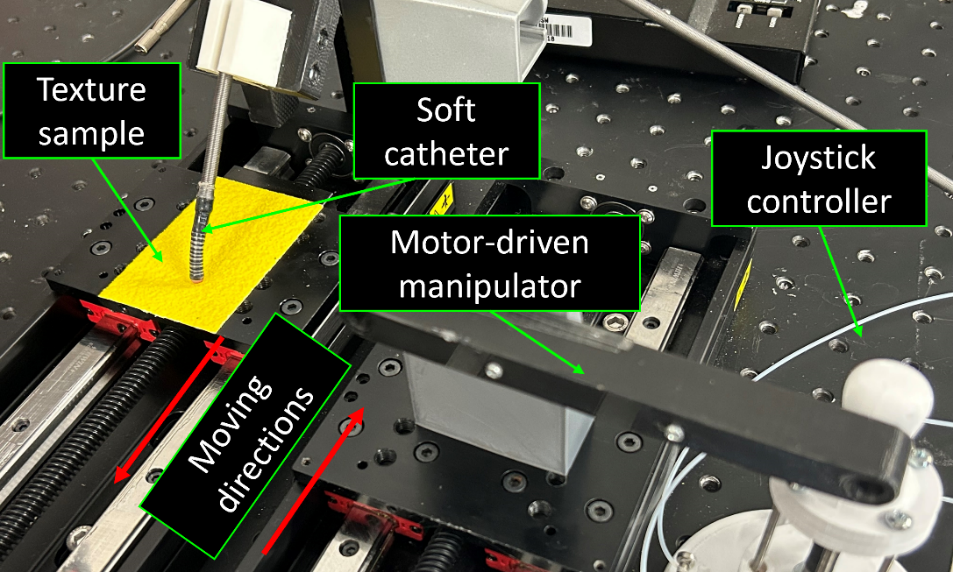


**Figure S14.** Experimental setup for detecting the texture of the sandpapers. The motor-driven manipulator moved the joystick controller to bend the SRA (activating SFSAs 2 and 4) touching the texture sample, which then was moved by the linear motor letting the catheter slide on it.

**Supplementary Note 5. Dynamic test**

Here, we report a dynamic test to assess the impact of the external environment on the control accuracy of the SFSA-based soft catheter. Specifically, water flow was manually increased from 0 to about 5 L.min^-1^ (measured by Atrato Series Ultrasonic Flow Meter, 0.1 L.min^-1^ – 20 L.min^-1^, Atrato), roughly simulating cardiac output, while the soft robotic catheter was activated with a consistent sinusoidal input motion (frequency 0.2 Hz) through the testing process (**Figure S15**). As a result, the internal pressures of the SFSAs were slightly changed which caused errors in the position of the catheter tip compared to the non-flow condition. The RMSEs have been computed with a maximum of 0.55 mm observed in the position on the Y-axis, which is about 5% of the motion range. The results demonstrate that external environmental factors adversely impact the system’s control accuracy. To better understand the environmental influence and mitigate these effects, future work will involve conducting more extensive dynamic tests (e.g., with higher flows and different directions) and exploring advanced compensation strategies (e.g., increasing the device’s stiffness).


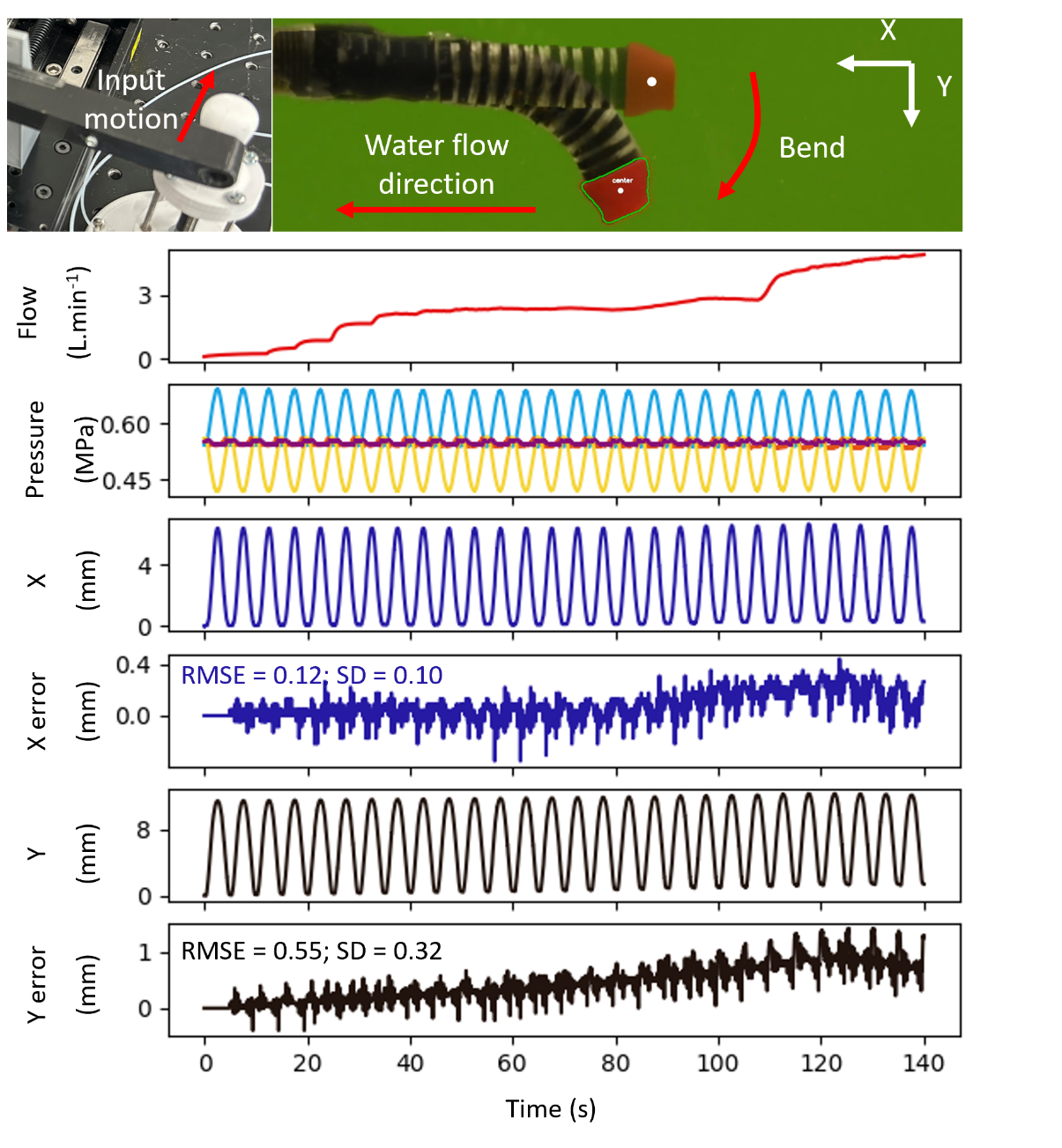


**Figure S15.** Dynamic test for the soft robotic catheter with computed errors (**Movie S12**).

# **Supplementary Note 6. Legends of the Supplementary Movies**

- **Movie S1**: Working principle, simulation, and prototype of Soft Fibrous Syringe Architecture (SFSA).
- **Movie S2**: Demonstrations of the soft robotic catheter powered by SFSA - *Controlling the soft robotic arm (SRA) using the desktop version with the robot’s state feedback.*
- **Movie S3**: Demonstrations of the soft robotic catheter powered by SFSA - *Controlling the soft robotic arm (SRA) using the desktop version with a higher sensitivity and the third DOF.*
- **Movie S4**: Demonstrations of the soft robotic catheter powered by SFSA - *Controlling the soft robotic arm (SRA) using the wearable version.*
- **Movie S5**: Demonstrations of controlling the continuum robot using an SFSA-based controller*.*
- **Movie S6**: Demonstrations of stiffness detection - *Passive sensing.*
- **Movie S7**: Demonstrations of surface mapping - *Passive sensing.*
- **Movie S8**: Demonstrations of touch sensing ability - *Passive sensing.*
- **Movie S9**: Demonstrations of touch sensing ability - *Active sensing.*
- **Movie S10**: Demonstrations of applying SFSA to different types of soft hydraulic artificial muscles/actuators.
- **Movie S11**: Demonstrations of reducing hand tremors.
- **Movie S12**: Dynamic test.

**References**

[1] C. C. Nguyen, T. Teh, M. T. Thai, P. T. Phan, T. T. Hoang, J. Davies, H.-P. Phan, C. H. Wang, N. H. Lovell, T. N. Do, *IEEE Transactions on Medical Robotics and Bionics* **2023**.

[2] M. Zhu, T. N. Do, E. Hawkes, Y. Visell, *Soft Robotics* **2020**, 7, 179.

[3] C. C. Nguyen, M. T. Thai, T. T. Hoang, J. Davies, P. T. Phan, K. Zhu, L. Wu, M. A. Brodie, D. Tsai, Q. P. Ha, *Sensors and Actuators A: Physical* **2023**, 357, 114380.

[4] A. Sedal, D. Bruder, J. Bishop-Moser, R. Vasudevan, S. Kota, *Journal of Mechanisms and Robotics* **2018**, 10, 024501.

[5] F. Connolly, C. J. Walsh, K. Bertoldi, *Proceedings of the National Academy of Sciences* **2017**, 114, 51.

[6] A. Gizzi, A. Pandolfi, M. Vasta, *Mechanics of Materials* **2016**, 92, 119.

[7] R. W. Ogden, *Courier Corporation*, **1997**.

[8] R. J. Webster III, B. A. Jones, *The International Journal of Robotics Research* **2010**, 29, 1661.

[9] C. Della Santina, A. Bicchi, D. Rus, *IEEE Robotics and Automation Letters* **2020**, 5, 1001.
